# Transcriptome Analysis of *Stephania yunnanensis* and Functional Validation of CYP80s Involved in Benzylisoquinoline Alkaloid Biosynthesis

**DOI:** 10.3390/molecules30020259

**Published:** 2025-01-10

**Authors:** Wenlong Shi, Qishuang Li, Xinyi Li, Linglong Luo, Jingyi Gan, Ying Ma, Jian Wang, Tong Chen, Yifeng Zhang, Ping Su, Xiaohui Ma, Juan Guo, Luqi Huang

**Affiliations:** 1 State Key Laboratory for Quality Ensurance and Sustainable Use of Dao-di Herbs, National Resource Center for Chinese Materia Medica, China Academy of Chinese Medical Sciences, Beijing 100700, China; shiwl031@163.com (W.S.); liqs556@163.com (Q.L.); lixinyi_cq@126.com (X.L.); linglongluo@163.com (L.L.); ganjingyi01@163.com (J.G.); xiaoma1110@126.com (Y.M.); jianwang2021@126.com (J.W.); chentong_biology@163.com (T.C.); yifengzhang06@126.com (Y.Z.); suping120@163.com (P.S.); huangluqi01@163.com (L.H.); 2 Yunnan Key Laboratory of Southern Medicinal Utilization, College of Chinese Materia Medica, Yunnan University of Chinese Medicine, Kunming 650500, China; maxiaohui1988@126.com

**Keywords:** aporphine alkaloids, biosynthetic gene clusters, biosynthesis, CYP80, *Stephania yunnanensis*, transcriptome

## Abstract

The medicinal plant *Stephania yunnanensis* is rich in aporphine alkaloids, a type of benzylisoquinoline alkaloid (BIA), with aporphine being the representative and most abundant compound, but our understanding of the biosynthesis of BIAs in this plant has been relatively limited. Previous research reported the genome of *S. yunnanensis* and preliminarily identified the norcoclaurine synthase (NCS), which is involved in the early stages of the BIA biosynthetic pathways. However, the key genes promoting the formation of the aporphine skeleton have not yet been reported. In this study, based on the differences in the content of crebanine and several other BIAs in different tissues, we conducted transcriptome sequencing of roots, stems, and leaves. We then identified candidate genes through functional annotation and sequence alignment and further analyzed them in combination with the genome. Based on this analysis, we identified three CYP80 enzymes (SyCYP80Q5-1, SyCYP80Q5-3, and SyCYP80G6), which exhibited different activities toward (*S*)- and (*R*)-configured substrates in *S. yunnanensis* and demonstrated strict stereoselectivity enroute to aporphine. This study provides metabolomic and transcriptomic information on the biosynthesis of BIAs in *S. yunnanensis*, offers valuable insights into the elucidation of BIA biosynthesis, and lays the foundation for the complete analysis of pathways for more aporphine alkaloids.

## 1. Introduction

*Stephania yunnanensis* is one of the source plants of the traditional Chinese medicine “Shanwugui”, which is a plant from the genus *Stephania* in the family Menispermaceae of the order Ranales [1]. Traditional Chinese medicine theory believes that *S. yunnanensis* has the effects of vomiting phlegm and foods, treating malaria, and detoxifying sores [2]. Previous chemical research has shown that the main active components of *S. yunnanensis* are four types of benzylisoquinoline alkaloids (BIAs), including 1-benzylisoquinolines (1-BIAs), aporphines, morphinans, and protoberberines [3,4,5] such as salutaridine, tetrahydropalmatine, roemerine, stephanine, and crebanine. Crebanine, a kind of aporphine, is a major active compound found in *S. yunnanensis* [6]. Recent studies have indicated that crebanine possesses neuroprotective [7], cardioprotective [8], anticancer [9,10], anti-inflammatory, and analgesic activities [11].

Since the extraction of BIAs from plants was an expensive process, and the content was also affected by the growth conditions of the plants, the low yield and variability limited the commercial development of BIAs. The complexity of chemical synthesis further hindered the large-scale production of BIAs. The demand for BIAs ultimately promoted the development of BIA metabolic engineering and synthetic biology. The biosynthetic pathways of BIAs were initiated with the enzymatic reaction of norcoclaurine synthase (NCS), which catalyzes the synthesis of norcoclaurine from dopamine and 4-hydroxyphenylacetic acid (4-HPAA) [12]. Subsequent biochemical transformations included hydroxylation, methylation, and isomerization processes involving an array of cytochrome P450s, methyltransferases, and reductases, such as norcoclaurine 6-*O*-methyltransferase (6OMT) [13], coclaurine *N*-methyltransferase (CNMT), (*S*)-*N*-methylcoclaurine 3′-hydroxylase (NMCH) [14,15], 3′-hydroxy-*N*-methylcoclaurine, and 4′-*O*-methyltransferase (4′OMT) [16]. These enzymes catalyzed the production of a series of crucial 1-BIA intermediates, including coclaurine (COC), *N*-methylcoclaurine (NMC), 3′-hydroxyl-*N*-methylcoclaurine (HNMC), and reticuline, which subsequently served as substrates for the biosynthesis of a diverse array of BIAs.

Aporphine alkaloids like crebanine are the major compounds in *S. yunnanensis*, which has an unclear biosynthetic pathway. The CYP80 family is an important group of genes in the biosynthetic pathway of BIAs, being involved in the hydroxylation of 1-BIA and subsequent reactions such as C-C coupling. Recent studies have discovered that corytuberine synthases (CTSs) like CjCYP80G2 from *Coptis japonica* [17] and StCYP80G6 [18] from *Stephania tetrandra* could catalyze C-C coupling of (*S*)-reticuline to type I aporphine (*S*)-corytuberine. Additionally, NnCYP80Q1 or StCYP80Q5 could catalyze C-C coupling of (*R*)-coclaurine and (*R*)-*N*-methylcoclaurine to the protoaporphines (*R*)-crotoflorine and (*R*)-glaziovine, two important precursors of type II aporphines [18,19]. Subsequently, the proaporphines crotoflorine and glaziovine undergo unknown oxidative rearrangements, followed by a series of hydroxylations and methylations and the formation of methylenedioxy bridges, leading to the production of type II aporphines, including crebanine and nelumboferine [18]. These CYP80s are considered to be the starting points of the aporphine biosynthetic pathways. However, no CYP80s with similar catalytic C-C coupling functions have been found in *S. yunnanensis*.

The combination of transcriptomics and metabolomics is currently the most common method for studying the biosynthesis and regulation of plants’ secondary metabolites [20,21,22,23]. To date, de novo transcriptomes of multiple BIA-producing plants have been reported, focusing on the Papaveraceae, Ranunculaceae, and Menispermaceae families within the Ranunculales order [24]. Papaveraceae plants have been a primary focus due to their unique accumulation of morphinan alkaloids [25,26,27,28]. As the pharmacological effects of other types of BIAs have been discovered, the transcriptomes of other BIA-producing plants have also been reported to enable more in-depth research into BIA biosynthesis. The genome of *S. yunnanensis* has been previously reported [29], providing valuable genomic data for investigating its BIA biosynthetic pathways. However, for a deeper understanding of BIA biosynthesis and its regulation, it is also necessary to have information on the gene expression levels and metabolite profiles. Unfortunately, there are currently no reported transcriptome data available for *S. yunnanensis*.

In this study, we determine the relative concentrations of four BIAs in different tissues of *S. yunnanensis* to investigate the differential accumulation patterns of these BIAs. We also generate a de novo transcriptome of *S. yunnanensis* and identified hundreds of genes potentially related to BIA biosynthesis. Subsequently, using differential expression analysis and phylogenetic analysis, we identify some candidate genes involved in BIA biosynthesis in *S. yunnanensis* and conduct genome-level analysis of them. We then validate the functions of the CYP80 genes of *S. yunnanensis* in vitro. Our research will contribute to further in-depth studies on BIA biosynthetic pathways and their evolution in *Stephania* species, aiming to elucidate the complete biosynthetic pathways of BIAs represented by crebanine.

## 2. Results

### 2.1. Determination of BIAs in S. yunnanensis

**Figure 1:**
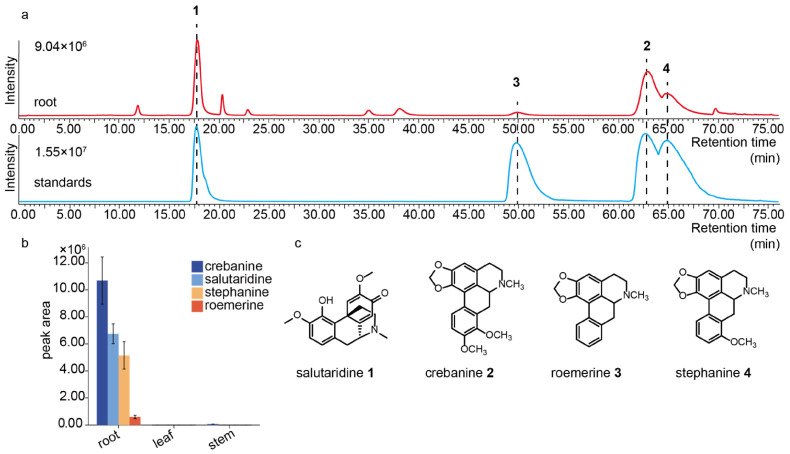
(**a**) Determination of BIAs in *S. yunnanensis*. LC-MS chromatogram of *S. yunnanensis* roots, with peaks of four compounds labeled with corresponding numbers. Compounds **1**, **2**, **3**, and **4** correspond to salutaridine, crebanine, roemerine, and stephanine. The concentration of all four chemical standards was 0.02 mM. (**b**) A bar chart of the relative content based on the average peak area, with four compounds represented by different colors. (**c**) The structures of the four main BIAs.

*S. yunnanensis* is a medicinal plant with a long history of use, with BIAs as its main active components [3,4]. Due to its strict environmental requirements and long growth cycle, it had not yet been cultivated on a large scale. Recent studies have reported the presence of various aporphine alkaloids in *S. yunnanensis* (Figure S1) [5]. We analyzed three tissues of *S. yunnanensis* using liquid chromatography-mass spectrometry (LC-MS) and researched the relative contents of salutaridine, roemerine, stephanine, and crebanine to investigate the accumulation patterns of these four representative BIAs in *S. yunnanensis* (Figure 1 and Figure S2). Surprisingly, we detected four representative BIAs in the roots, each corresponding to the purchased reference standards, but in the stems and leaves, there were hardly any or only minimal amounts detected (Figure S2). Furthermore, crebanine was the most abundant among the four compounds (Figure 1), which is consistent with previous studies [6]. Among these compounds, salutaridine was identified as a morphinan, another major compound reported in *S. yunnanensis* [6]. Additionally, roemerine and stephanine were considered important intermediates, along with the representative product crebanine, all of which belong to type II aporphines [5]. We also attempted to detect additional aporphines which have been reported in other *Stephania* species [5,6]. However, we did not detect magnoflorine, a representative type I aporphine, suggesting that *S. yunnanensis* may contain more type II aporphines, with type I aporphines being less prevalent. This result might explain why *S. yunnanensis* was primarily used for its tuberous roots in medicine rather than its aerial parts. However, under the current extraction and detection conditions, we did not find any representative bisbenzylisoquinoline cepharanthine, which contradicts another study that reported the presence of cepharanthine and other bisbenzylisoquinolines in the metabolome of *S. yunnanensis* [29]. In summary, the analysis of these compounds indicated that numerous structurally diverse BIAs are present in *S. yunnanensis*, specifically accumulating in the roots, with aporphines, represented by crebanine, being the most abundant.

### 2.2. Transcriptome Sequencing, Assembly, and Analysis of S. yunnanensis

To analyze the biosynthesis of aporphine in *S. yunnanensis* and ensure the accuracy and reproducibility of the results, we collected roots, stems, and leaves from three plants grown under similar conditions, totaling nine samples for transcriptome sequencing. Since *S. yunnanensis* is a wild species, we aimed to enhance the reliability and robustness of the results by considering the natural variation between individual plants. De novo assembly yielded 50,119 transcripts, with an N50 length of 2041 bp and an average length of 1681 bp (Table 1).

Benchmarking Universal Single-Copy Ortholog (BUSCO) assessment [30] indicated that the *S. yunnanensis* transcriptome contained 87.3% complete BUSCOs (Figure S3a and Table S1). Annotation was performed for all 50,119 (100%) transcripts across various databases, with an annotated transcript N50 length of 1518 bp (Table S2). Gene Ontology (GO) classification [31] was conducted to describe gene functions in three categories: cellular component, molecular function, and biological process (Figure S3b).

**Figure 2:**
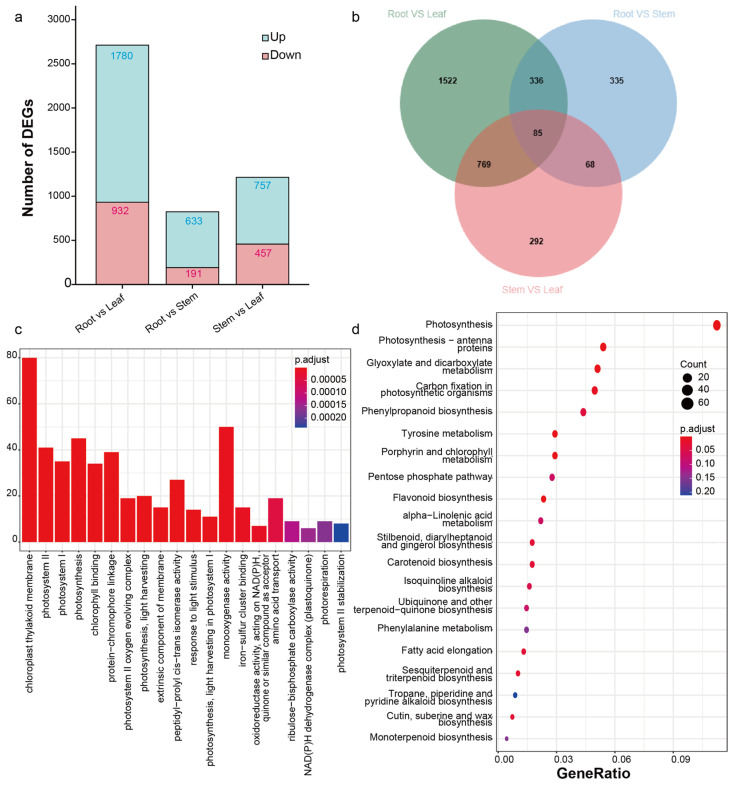
Functional annotation of DEGs. (**a**) DEGs of root vs. leaf, root vs. stem, and stem vs. leaf in *S. yunnanensis* and their Venn diagram (**b**), where circles in different colors represent the DEGs between different organ combinations. Green represents “root vs. leaf”, blue represents “root vs. stem”, and pink represents “stem vs. leaf”. The overlapping areas of two circles indicate DEGs common to two organ combinations, while the overlapping area of all three circles represents DEGs common to all three organ combinations. (**c**) GO enrichment analysis of DEGs in root vs. leaf. (**d**) Scatterplot of KEGG pathway enrichment of DEGs in root vs. leaf.

To investigate the distribution of assembled transcripts in each tissue, their relative expression levels were determined by calculating the fragment per kilobase of transcript per million fragments mapped (FPKM) values of the assembled transcripts. To compare the differentially expressed genes (DEGs) between roots and other tissues, we conducted a comparative analysis of the expression levels of the assembled transcripts (Figure 2). The results indicated that the number of DEGs between the roots and leaves was highest compared with the other groups, with 1780 upregulated and 932 downregulated (Figure 2a,b). To further analyze the DEGs between the roots and leaves, GO and KEGG clustering analyses were performed (Figure 2c,d and Figure S4). GO clustering revealed that most DEGs between the roots and leaves were classified into “monooxygenase activity-related” categories, except for photosynthesis-related categories (Figure 2c). On the other hand, KEGG clustering showed that a total of 685 DEGs were considered to be involved in secondary metabolism, with only a small number (11 DEGs, with 4 downregulated and 7 upregulated) clustered into the “isoquinoline alkaloid biosynthesis” category (Figure 2d).

### 2.3. Analysis of BIA Biosynthetic Genes

**Figure 3:**
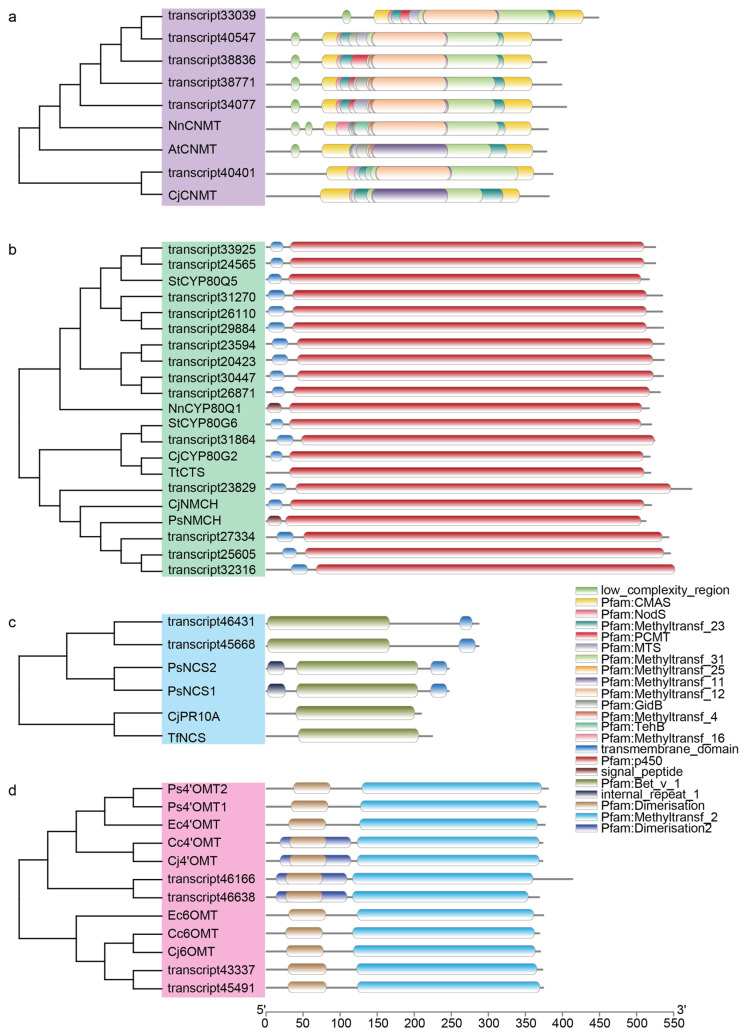
Phylogenetic and domain analysis of genes involved in BIA biosynthesis. Phylogenetic tree of functional genes and candidate transcripts from *S. yunnanensis*, constructed using the maximum likelihood method and protein domain analysis. (**a**) Reference CNMTs and candidate genes. (**b**) Reference P450s and candidate genes. (**c**) Reference NCSs and candidate genes. (**d**) Reference OMTs and candidate genes.

Apart from the reported SyNCS4 and SyNCS5 [29], the biosynthesis of BIAs in *S. yunnanensis* has not yet been elucidated. The early stages of the pathways, from dopamine and 4-HPAA to reticuline, were largely conserved among BIA-producing plants [32]. Analysis of the annotation results revealed a total of 141 OMTs, 117 NMTs, 45 NCSs, and 505 P450s (Tables S5–S8). In recent studies, CYP80s involved in BIA biosynthesis have gained increasing attention for their roles in the synthesis of 1-BIAs, as well as bisbenzylisoquinoline alkaloids and aporphine alkaloids [18,33]. Notably, among the P450s, 25 were further annotated into the CYP80 family, which are likely involved in BIA biosynthesis. To investigate the BIA biosynthesis in *S. yunnanensis*, we identified candidate genes with high similarity to previously reported BIA biosynthetic genes from *S. tetrandra* [20,21,34]. We selected sequences with homology levels greater than 55% and coverage greater than 90% as candidate genes, and a total of 26 candidate transcripts for the BIA biosynthesis were identified from the *S. yunnanensis* transcriptome. Phylogenetic analysis and domain analysis indicated that these candidate transcripts have structural similarities to the reference genes (Figure 3 and Table S3). The sequence alignment revealed that these candidate genes share similar sequences with the reference genes (Figures S5–S8). To analyze the expression patterns of these candidate transcripts in different tissues of *S. yunnanensis*, a heatmap was generated using the FPKM of the candidate genes (Figure S9). The heatmap showed that, except for NCS, 4′OMT, and CYP80G6, most candidate transcripts did not exhibit distinct differential expression patterns and maintained high expression levels across multiple tissue parts. This may explain why only a few DEGs were clustered into the “isoquinoline alkaloid biosynthesis” category. The disparity between gene expression and compound accumulation suggests that BIA compound transport might occur in *S. yunnanensis*. On the other hand, this disparity also suggests that the genes involved in the late stages of BIA biosynthesis might not be highly expressed only in the roots, an important consideration for gene screening.

**Figure 4:**
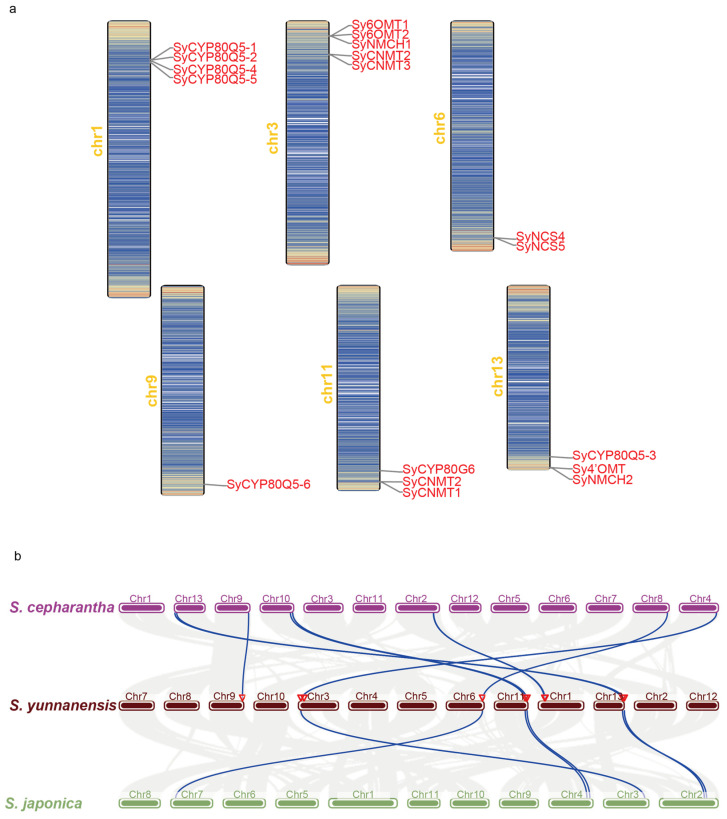
Chromosomal localization of *S. yunnanensis* gene and synteny analysis of the genomes of three *Stephania* species. (**a**) Chromosomal localization of candidate genes for BIA biosynthesis in *S. yunnanensis*. Pseudomolecules are represented as long bars, with colors indicating gene density: blue for low density and red for high density. (**b**) Synteny analysis of the genomes of three *Stephania* species, with the candidate genes in *S. yunnanensis* highlighted.

Furthermore, based on the reported *S. yunnanensis* genome [29], we examined the distribution and location of these candidate transcripts on the chromosomes (Figure 4a). Sequence alignment revealed that 26 candidate transcripts corresponded to 18 genes in genome. They were arranged in descending order of homology and named according to their respective functional genes before being mapped onto the chromosomes. Notably, among these candidate genes, one SyNMCH, two SyCNMTs, and two Sy6OMTs were located on pseudochromosome 3 in close proximity, suggesting that this region might form a BIA biosynthetic gene cluster (BGC). These were the genes involved in the three consecutive steps following NCS in the early stages of the BIA biosynthetic pathways. Similarly, another candidate gene, SyCYP80G6, co-localized with two SyCNMTs on pseudochromosome 11. Additionally, the candidate genes for CYP80Q5 were found on three different pseudochromosomes. Specifically, four were located on pseudochromosome 1, one was located on pseudochromosome 9, and another was co-localized with Sy4′OMT and SyNMCH2 on pseudochromosome 13. Among them, SyCYP80Q5-1 and SyCYP80Q5-2 shared over 98% of their identities, indicating they are tandem repeats, whereas they had 90% identity with SyCYP80Q5-3 (Figure S10). The other three genes, SyCYP80Q5-4, SyCYP80Q5-5, and SyCYP80Q5-6, had low identity with other SyCYP80Q5 genes, ranging from approximately 59% to 65%. These chromosomal locations preliminarily suggest that the BIA biosynthetic genes in *S. yunnanensis* may exhibit a certain degree of a clustered distribution. To investigate the distribution and association of these candidate genes in *Stephania* species, we conducted a synteny analysis using the reported genomes of two other plants: *S. japonica* and *S. cepharantha* (Figure 4b) [29]. Interestingly, the two tandemly duplicated genes of SyCYP80Q5 on chromosome 1 of *S. yunnanensis*, SyCYP80Q5-1 and SyCYP80Q5-2, had only one ortholog in *S. cepharantha* and none in *S. japonica*, but SyCYP80Q5-3 was conserved across the three species. Notably, the clusters observed in *S. yunnanensis* were present on different chromosomes in the three *Stephania* species. This indicates that the clustering patterns of BIA biosynthetic genes were conserved across the three species, suggesting that such clustering is likely conserved within *Stephania* as well. In summary, chromosome localization and synteny analysis have provided insights into the genomic-level associations of the BIA biosynthetic genes in *S. yunnanensis*.

### 2.4. Functional Verification and Phylogenetic Analysis of CYP80s

**Figure 5:**
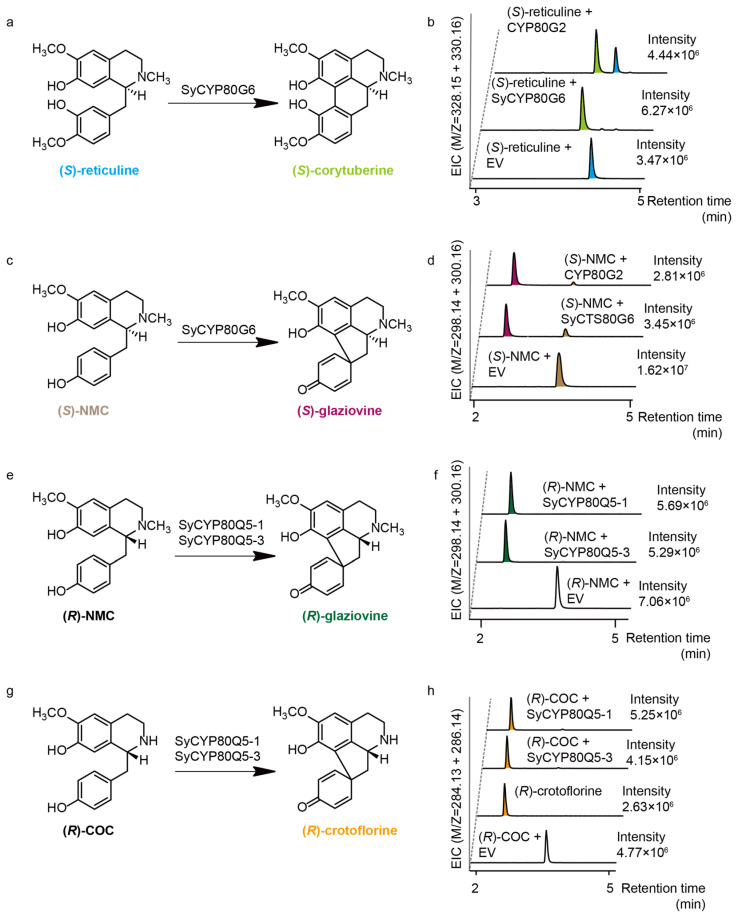
In vitro functional validation of the CYP80s from *S. yunnanensis*. SyCYP80G6 was responsible for catalyzing (*S*)-type substrates, and SyCYP80Q5 was responsible for catalyzing (*R*)-type substrates. The color of each product’s name corresponds to the colors of the chromatographic peaks. (**a**,**b**) SYCYP80G6 catalyzes the conversion of (*S*)-reticuline into (*S*)-corytuberine. (**c**,**d**) SyCYP80G6 catalyzes the conversion of (*S*)-NMC into (*S*)-glaziovine. (**e**,**f**) SyCYP80Q5-1 and SyCYP80Q5-3 catalyze the conversion of (*R*)-NMC into (*R*)-glaziovine. (**g**,**h**) SyCYP80Q5-1 and SyCYP80Q5-3 catalyze the conversion of (*R*)-COC into (*R*)-crotoflorine.

To elucidate the biosynthesis of the BIAs of *S. yunnanensis*, we conducted in vitro functional validation of the two key CYP80s involved in aporphine skeleton formation: CYP80G6 (CTS) and CYP80Q5. These enzymes specifically catalyzed the (*S*) or (*R*) types of 1-BIA substrates to produce the corresponding type I aporphines or type II aporphines (protoaporphines). The further rearrangement and modification of these intermediates ultimately led to the formation of aporphines such as crebanine. Due to the high degree of identity between SyCYP80Q5-1 and SyCYP80Q5-2, we cloned only SyCYP80Q5-1 and another four candidates—SyCYP80Q5-3, SyCYP80Q5-4, SyCYP80Q5-5, and SyCYP80Q5-6—as well as SyCYP80G6 and validated their functions in vitro. Recombinant plasmids containing these candidate genes were constructed and expressed in *Saccharomyces cerevisiae* (WAT11), in which the endogenous cytochrome P450 reductase was replaced by one from *Arabidopsis thaliana*. This is a commonly used eukaryotic expression strain for P450, allowing for in vitro functional validation without the need to reintroduce cytochrome P450 reductase. Microsomes extracted from the recombinant yeast were used for in vitro enzyme assays and were detected via LC-MS. We found that only SyCYP80Q5-1, SyCYP80Q5-3, and SyCYP80G6 exhibited catalytic functions (Figure 5, Figures S11 and S12). Specifically, SyCYP80G6 catalyzes the conversion of (*S*)-reticuline and (*S*)-NMC into (*S*)-corytuberine and (*S*)-glaziovine (Figure 5a–d and Figure S11a,c). Both SyCYP80Q5-1 and SyCYP80Q5-3 were capable of catalyzing the conversion of (*R*)-COC and (*R*)-NMC into the aporphine alkaloids (*R*)-crotoflorine and (*R*)-glaziovine (Figure 5e–h, Figures S11b and S12). Furthermore, the catalytic efficiency of SyCYP80Q5-1 was slightly higher than that of SyCYP80Q5-3 when using either (*R*)-COC or (*R*)-NMC as substrates (Figure 5f,h).

**Figure 6:**
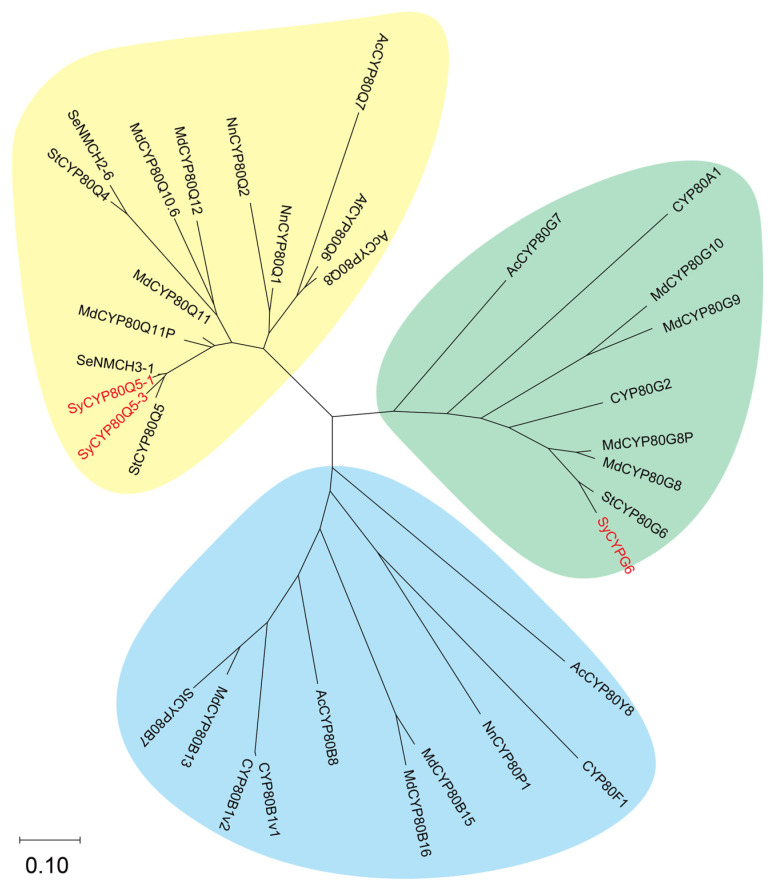
Phylogenetic tree of CYP80s. The red font represents P450s identified in this study.

Additionally, we discussed the activity of SyCYP80Q5-1 and SyCYP80G6 on a broader range of BIA substrates and other configurations (Figures S13 and S14). As expected, SyCYP80Q5 and SyCYP80G6 exhibited strict substrate specificity and stereoselectivity, which was consistent with the results of other CYP80s [18]. We performed a phylogenetic analysis of the CYP80 family, and the results showed that the three SyCYP80s clustered within the CYP80 family (Figure 6) were distributed across various clades of the CYP80 family. The functions of these enzymes matched the previously reported enzymes [18], indicating that these three enzymes play similar roles in BIA biosynthesis in *S. yunnanensis*.

## 3. Discussion

The biosynthetic pathways of BIAs have not been fully elucidated, mainly due to the complex transformations from 1-BIAs to various types of BIA scaffolds and the subsequent modifications, including C-C coupling, C-O coupling, hydroxylation, and methylation [18,35]. This study reported the de novo transcriptome of *S. yunnanensis*, and through combined analysis of the transcriptome, metabolome, and genome, we identified 18 candidate genes involved in BIA biosynthesis in *S. yunnanensis*. The functions of three CYP80s were validated in vitro; they specifically catalyzed the C-C coupling of (*S*)- or (*R*)-configured 1-BIA substrates, resulting in the formation of aporphines or protoaporphines. This is an important step in the biosynthetic pathways of aporphines [18].

In medicinal plants where roots or rhizomes are used, the active components are often highly concentrated in the underground parts, with corresponding functional genes and regulatory factors showing significantly higher expression levels compared with the aboveground parts [23,36,37,38,39,40]. In this study, we analyzed the representative BIAs of *S. yunnanensis* and found that the major active component, crebanine, and three other BIAs were highly accumulated in the roots, similar to other root-based medicinal plants. Notably, no 1-BIAs were detected in the metabolomes of any tissues, possibly due to their complete consumption or concentrations below the detection limit. Furthermore, under the extraction and detection conditions of this study, the representative bisbenzylisoquinoline alkaloid, cepharanthine, was not detected.

Then, we sequenced the transcriptomes of *S. yunnanensis* using next-generation sequencing, followed by de novo assembly and functional annotation. Previous studies have shown that P450s are involved in almost all biosynthetic pathways of BIAs, specifically CYP80, which participates in hydroxylation and C-O and C-C coupling reactions [18,33,35,41]. CYP719 catalyzed the formation of the methylenedioxy bridge [18,42,43,44], a characteristic group of many active BIA components, including crebanine. We identified 25 transcripts annotated as “CYP80”, and 11 were annotated as “CYP719” in the de novo transcriptome of *S. yunnanensis*. Through annotation and sequence alignment, we identified 26 candidate transcripts involved in 10 steps of the BIA biosynthetic pathways. The heatmap showed that, except for a few genes, the majority were not specifically expressed in the roots but were highly expressed across multiple tissues. This pattern of metabolite accumulation and differential gene expression levels, while uncommon, is not unprecedented in BIA-producing plants. Similar mechanisms involving transporter proteins facilitating the movement of compounds within a plant have been reported previously in *C. japonica* [45,46] and *P. somniferum* [47]. We speculate that similar mechanisms may also exist in *S. yunnanensis*, facilitating the internal transfer of compounds through processes such as endocytosis and exocytosis. In summary, the transcriptome data obtained in this study are valuable for elucidating the biosynthetic pathways of BIAs, including crebanine.

BGCs, composed of tightly arranged genes which collectively participate in the biosynthesis of specific metabolites, allow organisms to coordinate and efficiently regulate gene expression and synergistic metabolic reactions [48]. With the reported high-quality genome of *S. yunnanensis* [29], this study also examined the chromosomal localization of the 18 candidate genes and found a tendency for several candidate genes to cluster. Further collinearity analysis showed that this clustering phenomenon is conserved among different *Stephania* species, indicating important functions in their evolutionary processes. Notably, the tandem duplication of SyCYP80Q5-1 and SyCYP80Q5-2 on chromosome 1 of *S. yunnanensis* was not conserved among different species, whereas another SyCYP80Q5-3 gene on chromosome 13 was widely conserved. This might explain why all *Stephania* species can produce aporphines and protoaporphines but differ in the types and amounts they contain. Furthermore, the number of CYP80Q5 copies in *S. yunnanensis* was significantly higher than CYP80G6, which is consistent with the finding that there are more type II aporphines present in *S. yunnanensis*. In conclusion, the differences and conservation of the BIA biosynthetic pathways in *Stephania* suggest that there are still variations within the genus. This may explain why there are significant differences in the types and contents of BIAs among different *Stephania* species.

As an important gene family in the BIA biosynthetic pathways (CYP80), this study further validated the functions of three CYP80s from *S. yunnanensis* in vitro, showing that they all had the ability to catalyze substrate C-C coupling with configurational selectivity. In particular, SyCYP80G6 specifically catalyzed the production of corresponding aporphines or protoaporphines from (*S*)-type substrates, where SyCYP80Q5-1 and SyCYP80Q5-3 specifically catalyzed the production of corresponding protoaporphines, the precursor of type II aporphines, from (*R*)-type substrates. The functions of CYP80 are complex and diverse, and there might be some evolutionary correlation. It is noteworthy that although SyCYP80G6 can also catalyze (*S*)-type substrates to produce aporphines and protoaporphines, similar to other CYP80Gs, we did not detect any representative reported (*S*)-type aporphines, such as magnoflorine or mecambroline, in *S. yunnanensis*. However, the expression level of SyCYP80G6 is not low. This discrepancy between metabolites and gene expression suggests that there may be mechanisms in *S. yunnanensis*’s aporphine biosynthesis which we have yet to understand. It is possible that the metabolic flux is diverted toward (*R*)-reticuline, leading to the biosynthesis of (*R*)-type aporphines, morphinans, and protoberberines.

In conclusion, the combined approach of metabolite analysis and transcriptome sequencing identified 26 candidate transcripts responsible for the biosynthesis of BIAs, including crebanine in *S. yunnanensis*. These genes might be responsible for BIA biosynthesis in *S. yunnanensis*. Furthermore, genome analysis of the 18 candidate genes showed clustering on chromosomes, suggesting the presence of related BGCs, and collinearity analysis indicated that these BGCs are conserved among different *Stephania* species. Finally, we identified three CYP80s from the *S. yunnanensis* transcriptome data as being involved in BIA biosynthesis using in vitro enzymatic reactions. Overall, our work provides valuable genetic information on *S. yunnanensis* and reveals the biosynthesis of BIAs in this medicinal plant. Additionally, the CYP80s we reported will offer further insights and suggestions for the evolution, structure, and functional studies of the CYP80 family. Screening for functional gene components involved in the BIA biosynthetic pathway across different species will also contribute to the synthetic biology of BIAs, providing more options for the heterologous production of these compounds.

## 4. Materials and Methods

### 4.1. Plant Materials, Chemicals, Reagents, and Strains

The *S. yunnanensis* plant samples were collected from Yunnan University of Traditional Chinese Medicine (Kunming, China) were kindly provided by Dr. Xiaohui Ma.

Authentic standards of (*S*)-*N*-methylcoclaurine and (*R*)-*N*-methylcoclaurine were obtained through commercial chemical synthesis by WuXi LabNetwork. The (*S*)-norcoclaurine, (*R*)-norcoclaurine, (*S*)-coclaurine, and (*R*)-coclaurine were derived from the chiral separation of commercial racemic standard. The racemic norcoclaurine and coclaurine were purchased from Baoji Herbest Bio-Tech Co., Ltd. (Baoji, China). Other standards were purchased from Shanghai yuanye Bio-Technology Co., Ltd. (Shanghai, China). The (*R*)-crotoflorine was prepared as described in a previous work [18].

The YPD medium was composed of 20 g L^−1^ peptone (OXOID, Thermo Fisher Scientific Inc., Nanjing, China), 10 g L^−1^ yeast extract (OXOID, Thermo Fisher Scientific Inc, Nanjing, China), and 20 g L^−1^ glucose (Beijing Solarbio Science & Technology Co., Ltd., Beijing, China), serving as a standard medium for cultivating yeast and preparing competent cells. YPL medium, used to induce gene expression in yeast, uses galactose (Beijing Solarbio Science & Technology Co., Ltd., Beijing, China) instead of glucose compared with YPD medium. Synthetic dropout minus uracil medium (SD-Ura, Beijing FunGenome Technology Co., Ltd., Beijing, China) with 20 g L^−1^ glucose was employed to select positive colonies transformed with the pESC-Ura vector. For plate preparation, agar (Beijing Dingguo Changsheng Bio-Technology Co., Ltd., Beijing, China) at a concentration of 20 g L^−1^ was added as necessary.

The WAT11 yeast strain with endogenous cytochrome P450 reductase replaced by that from *Arabidopsis thaliana*, which was maintained by this laboratory [49]. The pESC-Ura was purchased from Agilent Technologies, Inc., Santa Clara, CA, USA. The *Escherichia coli* Trans1 T1 strain from TransGen Biotech Co., Ltd. (Beijing, China) was used for routine plasmid assembly.

### 4.2. Alkaloid Extraction and Composition Analysis

First, 50 mg of root, stem, and leaf freeze-dried powders of *S. yunnanensis* were mixed with 2 mL of methanol, and then we sonicated the extracts for 0.5 h. After centrifugation, the supernatants were filtered with a nylon syringe filter (0.22 μm) [50]. Quantitative analysis was conducted on a UPLC-QTOF-MS system (Waters Corporation, Milford, CT, USA). The Acquity UPLC was carried out using a T3 column (Waters Technologies, 2.1 mm × 100 mm, 2.7 μm particle size) at 38 °C. The mobile phases consisted of eluent acetonitrile (A) and 0.1% aqueous formic acid (B) with a flow rate of 0.1 mL min^−1^ and the following optimized linear gradient elution program: 5–12% A from 0.0 min to 1.0 min, 12% A from 1.0 min to 15.0 min, 12–17% A from 15.0 min to 16.0 min, 17% A from 16.0 min to 66.0 min, 17–90% A from 66.0 min to 67.0 min, 90% A from 67.0 min to 72.0 min, 90–5% A from 72.0 min to 72.5 min, and 5% A from 72.5 min to 76.0 min. The Acquity UPLC system was coupled to a Waters Xevo G2-S QTOF mass spectrometer equipped with electrospray ionization (ESI). The instrument was operated in positive ion mode to perform full scan monitoring in the range of 50–800 *m*/*z*. The other operating parameters were set as follows: a capillary voltage of 0.5 kV; sample cone voltage of 40 V; extraction cone voltage of 4 V; source temperature of 100 °C; desolvation temperature 300 °C; and desolvation gas flow of 800 L h^−1^. The trap collision energy of low-energy function was set at 6 eV, while the ramp trap collision energy of high-energy function was set at 30–50 eV [51]. Data acquisition and processing were performed using MassLynx software, version 4.2.

### 4.3. Transcriptome Analysis

*S. yunnanensis* RNA was extracted using an R6827 Plant RNA Kit (Omega Bio-tek, Inc., Norcross, GA, USA) according to the manufacturer’s protocol. The RNA was quality-checked using Nanodrop, Qubit, and gel electrophoresis. Once the RNA samples were verified to be of acceptable quality, they were randomly fragmented using a Covaris sonicator. The entire library preparation process then involved end repair, A-tailing, adapter ligation, purification, and PCR amplification. Specifically, fragmented mRNA was used as a template with random oligonucleotides as primers to synthesize the first strand of cDNA in an M-MuLV reverse transcriptase system. The RNA strand was degraded with RNaseH, and the second strand of cDNA was synthesized using DNA polymerase I with dNTPs as substrates. The double-stranded cDNA underwent end repair, A-tailing, and sequencing adapter ligation. AMPure XP beads were used to select cDNA fragments of approximately 250–300 bp. After PCR amplification, the library was purified again using AMPure XP beads. Quality control of the library involved quantification with a Qubit 2.0 fluorometer, followed by size verification with an Agilent 2100 bioanalyzer. Once the insert size met our expectations, the effective library concentration was accurately quantified using qRT-PCR (with the effective library concentration exceeding 2 nM). The library preparation kit used was the NEBNext^®^ Ultra™ RNA Library Prep Kit for Illumina^®^, and sequencing was performed on an Illumina Novaseq 6000 platform.

To obtain comprehensive functional information of the transcripts, functional annotation was performed using seven databases, including Nr [52], Pfam [53], Uniprot [54], KEGG [55], GO [31], KOG/COG [56], and PATHWAY [55]. Functional annotation primarily employed two methods: sequence similarity search and motif similarity search. For the sequence similarity search, the protein sequences encoded by the transcripts were compared with existing protein databases, such as Uniprot, Nr [52], and the metabolic pathway database KEGG [55], using diamond BLASTp (version: 2.0.6.144; parameters: e-value 1 × 10^−5^) [57] to obtain functional information and potential metabolic pathway information. KEGG annotation was performed using KOBAS (version: 3.0) [58] to associate with KEGG ORTHOLOGY and PATHWAY. The Uniprot [54] database records the correspondence between each protein family and the functional nodes in Gene Ontology [31], allowing prediction of the biological functions executed by the protein sequences encoded by the transcripts. Based on the associations between databases, KOG/COG [56] annotation results were obtained, followed by classification statistics and plotting for KOG/COG [56]. For the motif similarity search, proteins typically consist of one or more functional regions, commonly referred to as domains. The different combinations of these domains result in a variety of proteins. Thus, identifying protein domains is crucial for analyzing protein functions. Domain prediction was performed using hmmscan (version: 3.3.2; parameters: e-value = 0.01) [59] to obtain the conserved sequences, motifs, and domains of the proteins. The Pfam [53] database is a large collection of protein families based on multiple sequence alignments and hidden Markov models.

The expression levels of the transcripts were assessed by calculating their FPKM values. If the samples all contained biological replicates, then we used DESeq2 for differential expression analysis; otherwise, we used edgeR. Herein, the differentially expressed genes (DEGs) were screened with thresholds where the significance levels were for corrected values of padj < 0.05 and |log2FoldChange| > 1. We performed GO enrichment and KEGG pathway enrichment analysis on the DEGs with padj < 0.05, which was considered significantly enriched among the DEGs.

### 4.4. Analysis of Candidate Genes in the BIA Biosynthetic Pathways

To identify the candidate genes associated with the BIA biosynthetic pathways of *S. yunnanensis*, local BLASTp by BioEdit, version 7.1.3.0 [60] was performed against *S. yunnanensis* sequences using query sequences of BIA-producing plants obtained from GenBank databases. The resulting transcripts with high degrees of identity were selected as candidate genes. A heatmap was generated using Tbtools, version 2.142 with row scaling. Based on the reported genome of *S. yunnanensis* [29], we studied the chromosomal localization of these candidate genes and conducted synteny analysis of three species using TbTools.

Based on the reference genes of BIA-producing plants and the obtained candidate genes, phylogenetic analysis was conducted using MEGA11 [61] through maximum likelihood estimation.

### 4.5. Cloning of Candidate Genes and Eukaryotic Expression of Recombinant Plasmids

All candidate genes were amplified from *S. yunnanensis* root or leaf cDNA using the primers listed in the Supplementary Materials (Table S4) and PrimeSTAR^®^ Max DNA Polymerase (Takara Co., Ltd., Tokyo, Japan) according to the manufacturer’s instructions. All CYP450 genes were purified and constructed into the pESC-Ura vector (digested with *BamHI*) using a Gibson Assembly Kit (TransGen Biotech Co., Ltd., Beijing, China). The recombinant vectors were transformed into competent *E. coli* Trans1 T1 and identified using colony PCR (TransGen Biotech Co., Ltd., Beijing, China) and Sanger sequencing (Beijing Ruibiotech Co., Ltd., Beijing, China). The identified positive recombinant plasmids were extracted from Trans1 T1 using a plasmid purification kit (Magen Biotechnology Co., Ltd., Guangzhou, China).

The pESC-Ura recombinant plasmids carrying candidate CYP450 genes were each transformed into WAT11 using the Frozen-EZ Yeast Transformation II Kit (Zymo Research Corporation, Irvine, CA, USA). WAT11 transformed with empty pESC-Ura was employed as a control. The positive transformants were screened on an SD-Ura medium containing 20 g L^−1^ glucose. The positive clones were incubated with shaking at 30 °C for 20–24 h until the OD600 reached 2–3. Cells were centrifuged to remove the SD-Ura medium. The cells were then resuspended in YPL induction medium and grown overnight at 30 °C to induce recombinant protein expression.

### 4.6. Microsome Extraction, Enzymatic Activity Assay, and LC-MS Analysis

Microsomes of recombinant yeast were prepared as previously described [62], the method for which was proven to be successful for such previous characterization. In vitro activity assays were performed in a 250 μL reaction system which included 100 mM Tris-HCl (pH of 7.5, Sangon Biotech (Shanghai) Co., Ltd., Shanghai, China), 500 μM NADPH (Beijing Solarbio Science & Technology Co., Ltd., Beijing, China), 0.1 g microsomal protein, and 20 μM of the substrate. The reactions were incubated at 30 °C for 2 h with shaking at 180 rpm, and then 5 μL of ammonium hydroxide was added to the reaction system to set the pH level to about 10 before extraction with 250 μL of ethyl acetate. After the ethyl acetate solution of the reaction product was concentrated to dryness, 150 μL of methanol was added to redissolve it before being centrifuged at 20,000× *g* for 15 min prior to LC-MS analysis. All enzymatic reaction products were detected using a UPLC-QTOF-MS system (Waters Corporation, Milford, CT, USA). The Acquity UPLC was carried out as previously described. The linear gradient elution program was 5–30% A from 0.0 min to 6.0 min, 30–60% A from 6.0 min to 8.0 min, 60–90% A from 8.0 min to 8.5 min, 90% A from 8.5 min to 9.5 min, 90–5% A from 9.5 min to 10.0 min, and 5% A from 10 min to 11 min. Data acquisition and processing were performed using MassLynx software [18].

## Figures and Tables

**Table 1:** Assembly results of de novo transcriptome of *S. yunnanensis*.

Item	Number
Seq. Num.	50,119
Seq. Base (bp)	91,278,165
N50 (bp)	2041
Max Length (bp)	7046
Min Length (bp)	108
Average Length (bp)	1821.23
Mean Length (bp)	1681.00

## Data Availability

The original contributions presented in this study are included in the article or Supplementary Materials. Further inquiries can be directed toward the corresponding author.

## Supplementary Materials

The following supporting information can be downloaded at https://www.mdpi.com/article/10.3390/molecules30020259/s1. Figure S1: The two types of aporphines in *S. yunnanensis*. Figure S2: LC-MS chromatograms of *S. yunnanensis* stems and leaves, where the concentration of all four chemical standards was 0.02 mM. Figure S3: Analysis of de novo transcriptome. (a) BUSCO evaluation results for the de novo transcriptome of *S. yunnanensis.* (b) GO clustering analysis of the de novo transcriptome of *S. yunnanensis*. Figure S4: GO and KEGG pathway enrichment of DEGs of root vs. stem and stem vs. leaf comparisons. Here, SyR, SyS, and SyL represent the roots, stems, and leaves of *S. yunnanensis*, respectively. Figure S5: Sequence alignment of candidate CNMTs and reference genes. Figure S6: Sequence alignment of candidate CYP80s and reference genes. Figure S7: Sequence alignment of candidate NCSs and reference genes. Figure S8: Sequence alignment of candidate OMTs and reference genes. Figure S9: Expression patterns of candidate transcripts involved in the biosynthesis of BIAs in *S. yunnanensis*. Putative biosynthetic pathway for BIAs and analysis of gene expression patterns related to BIAs in *S. yunnanensis*. The expression levels in different samples are indicated by small squares in different colors. The colors indicate the expression levels, with blue representing low expression and red representing high expression. From left to right: leaf 1, leaf 2, leaf 3, stem 1, stem 2, stem 3, root 1, root 2, and root 3. The pathway for type I aporphines is highlighted in blue, while the pathway for type II protoaporphines is highlighted in red. Figure S10: Sequence alignment of SyCYP80Q5-3, SyCYP80Q5-1, and StCYP80Q5. Figure S11: High-resolution MS/MS spectrum fragmentation of products catalyzed by CYP80s, including (a) comparison between the products of SyCYP80G6 and CYP80G2 catalyzing (*S*)-reticuline, (b) comparison between the two products of SyCYP80Q5-1 and SyCYP80Q5-3 catalyzing (*R*)-COC and (*R*)-NMC, and (c) comparison between the products of SyCYP80G6 and CYP80G2 catalyzing (*S*)-NMC. Figure S12: (a) Comparison between the products of SyCYP80Q5-1 and StCYP80Q5 catalyzing (*R*)-NMC and (b) comparison between (*R*)-crotoflorine standard and the products of SyCYP80Q5-1. Figure S13: Reaction of SyCYP80G6 with NMC. Figure S14: Reaction of SyCYP80G6 with NMC and COC. Table S1: BUSCO evaluation results. Table S2: Annotation results of the de novo transcriptome of *Stephania yunnanensis*. Table S3: Functional genes involved in the biosynthesis pathways of BIAs. Table S4: The PCR primers used in this study.
