# Supplementary material for: Transcriptome Analysis of Stephania yunnanensis and Functional Validation of CYP80s Involved in Benzylisoquinoline Alkaloid Biosynthesis
Source: Molecules. 2025 Jan 10;30(2):259. doi: 10.3390/molecules30020259 (PMC11767795; doi:10.3390/molecules30020259)
Supplement: Supplementary file 1 [file molecules-30-00259-s001.zip › Supplementary file1.docx]

| Item | Number | Percent |
| --- | --- | --- |
| Complete BUSCOs (C) | 371 | 87.3% |
| Complete and single-copy BUSCOs (S) | 121 | 28.5% |
| Complete and duplicated BUSCOs (D) | 250 | 58.8% |
| Fragmented BUSCOs (F) | 12 | 2.8% |
| Missing BUSCOs (M) | 42 | 9.9% |
| Total BUSCO groups searched | 425 | 100% |

**Table S1** BUSCO evaluation results.

| Item | Count | Percentage |
| --- | --- | --- |
| All | 50,119 | 100.00% |
| Annotation | 45,550 | 90.88% |
| Uniprot | 45,226 | 90.24% |
| Pfam | 40,599 | 81.01% |
| GO | 33,142 | 66.13% |
| KEGG | 24,216 | 48.32% |
| Pathway | 13,959 | 27.85% |
| KOG | 6,143 | 12.26% |
| Nr | 45,236 | 90.26% |

**Table S2** Annotation results of the *de novo* transcriptome of *Stephania yunnanensis*.

| Gene | Source | Accession |
| --- | --- | --- |
| Cj6OMT | *Coptis japonica* | BAB08004.1 |
| NnCNMT | *Nelumbo nucifera* | AXJ91467.1 |
| Cc6OMT2 | *Coptis chinensis* | AXC09386 |
| AtCNMT | *Arabidopsis thaliana* | AAM65762.1 |
| Ec6OMT | *Eschscholzia californica* | BAM37634.1 |
| CjBBE | *Coptis japonica* | BAM44344.1 |
| EcBBE | *Eschscholzia californica* | AAC39358.1 |
| CjNMCH | *Coptis japonica* | BAB12433.1 |
| PsNMCH | *Papaver somniferum* | AAF61400.1 |
| Cj4'OMT | *Coptis japonica* | Q9LEL5.1 |
| Cc4'OMT | *Coptis chinensis* | ABY75613.1 |
| Ec4'OMT | *Eschscholzia californica* | BAM37633.1 |
| Ps4'OMT1 | *Papaver somniferum* | Q7XB11.1 |
| Ps4'OMT1 | *Papaver somniferum* | Q7XB10.1 |
| CjCYP80G2 | *Coptis japonica* | BAF80448.1 |
| TtCTS | *Thalictrum thalictroides* | KAF5177347.1 |
| NnCYP80Q1 | *Nelumbo nucifera* | XP_010253990.1 |
| PsRNMT | *Papaver somniferum* | A0A1C9U5X5.1 |
| PsSLS | *Papaver somniferum* | ABR14720.1 |
| StCYP80G6 | *Stephania tetrandra* | (Li et al. 2024) |
| StCYP80Q5 | *Stephania tetrandra* | (Li et al. 2024) |
| CjPR10A | *Coptis japonica* | A2A1A1.2 |
| TfNCS | *Thalictrum flavum* | AAR22502.1 |
| PsNCS1 | *Papaver somniferum* | Q4QTJ2.1 |
| PsNCS2 | *Papaver somniferum* | Q4QTJ1.1 |
| CjCNMT | *Coptis japonica* | BAB71802.1 |

**Table S3** Functional genes involved in the biosynthesis pathways of BIAs.

| Primer | Sequence |
| --- | --- |
| SyCYP80Q5-1-F | ATGGCAGCAGCGC |
| SyCYP80Q5-1-R | TTATATAGGTAGATGAGATGTGATG |
| SyCYP80Q5-3-R | ATGGCAGCAGTACTACTACT |
| SyCYP80Q5-3-R | TCAGAATATGTGATTGAGTTTGGG |
| SyCYP80G6-R | ATGGAAGCAGTAGCAGC |
| SyCYP80G6-R | TTATGCATGCGGAATTCTCAC |

**Table S4** The PCR primers used in this study.


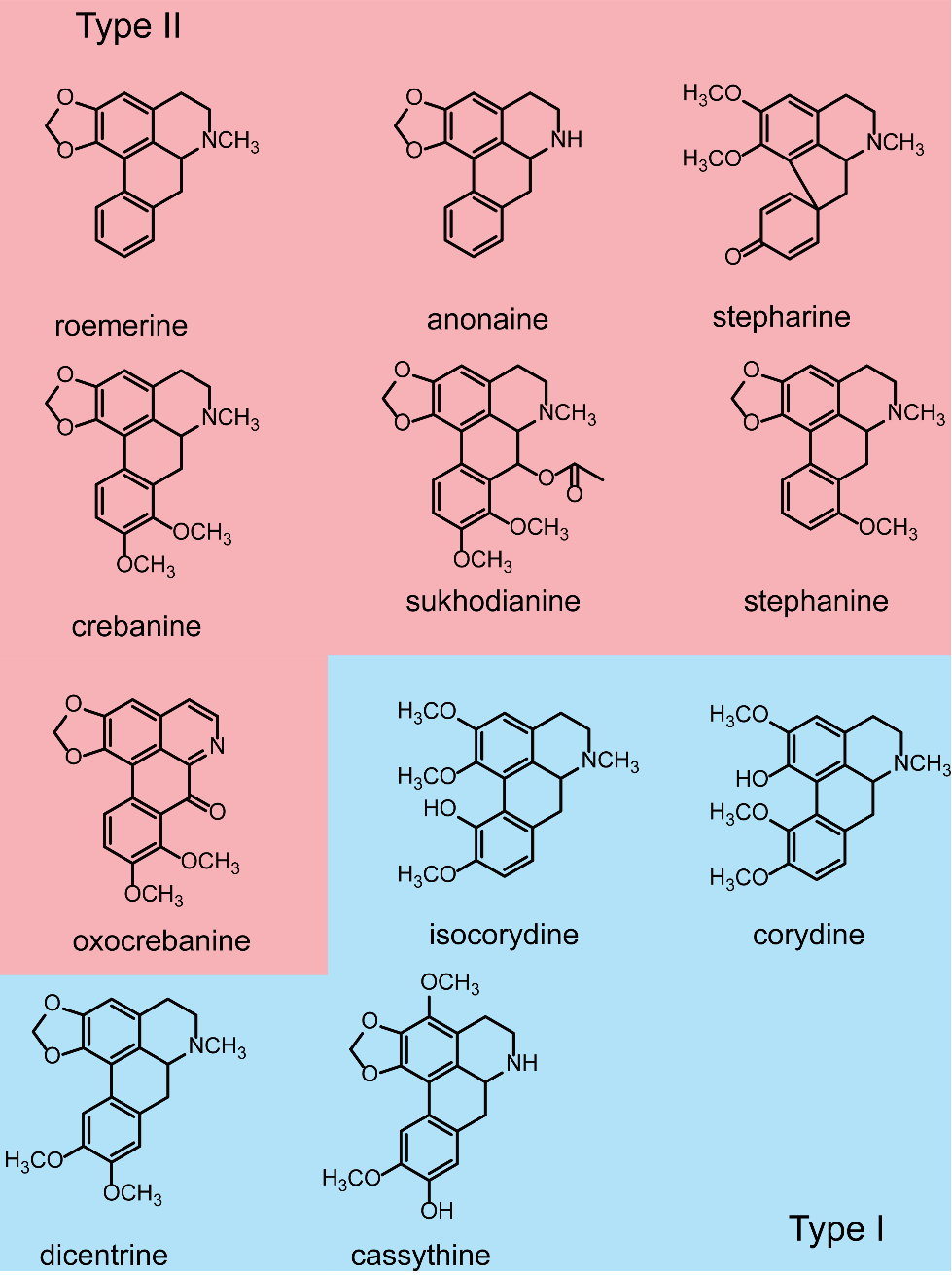


**Fig. S1** The two types of aporphines in *S. yunnanensis*.


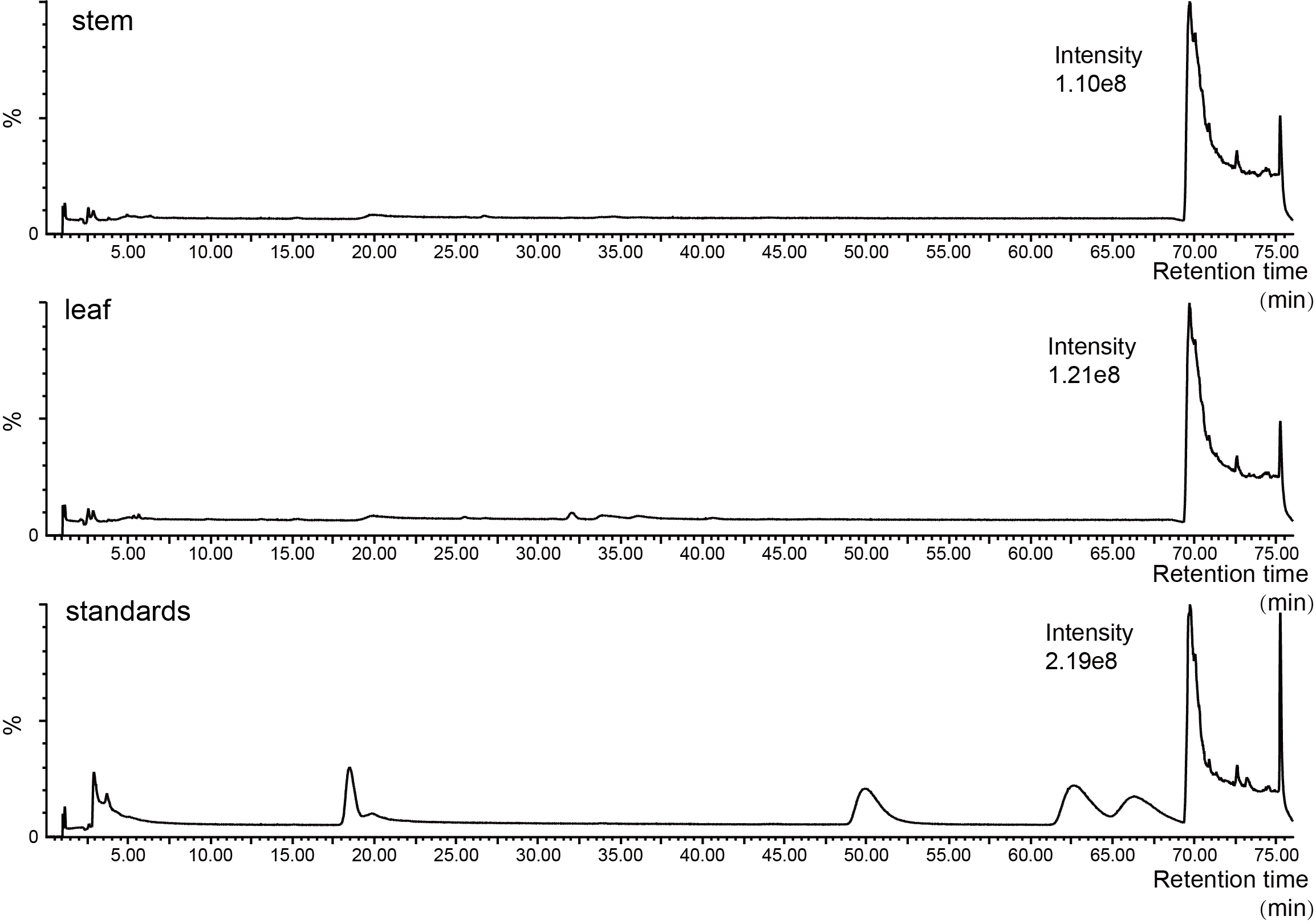


**Fig. S2** LC-MS chromatograms of *S. yunnanensis* stem and leaf, the concentration of all four chemical standards was 0.02 mM.


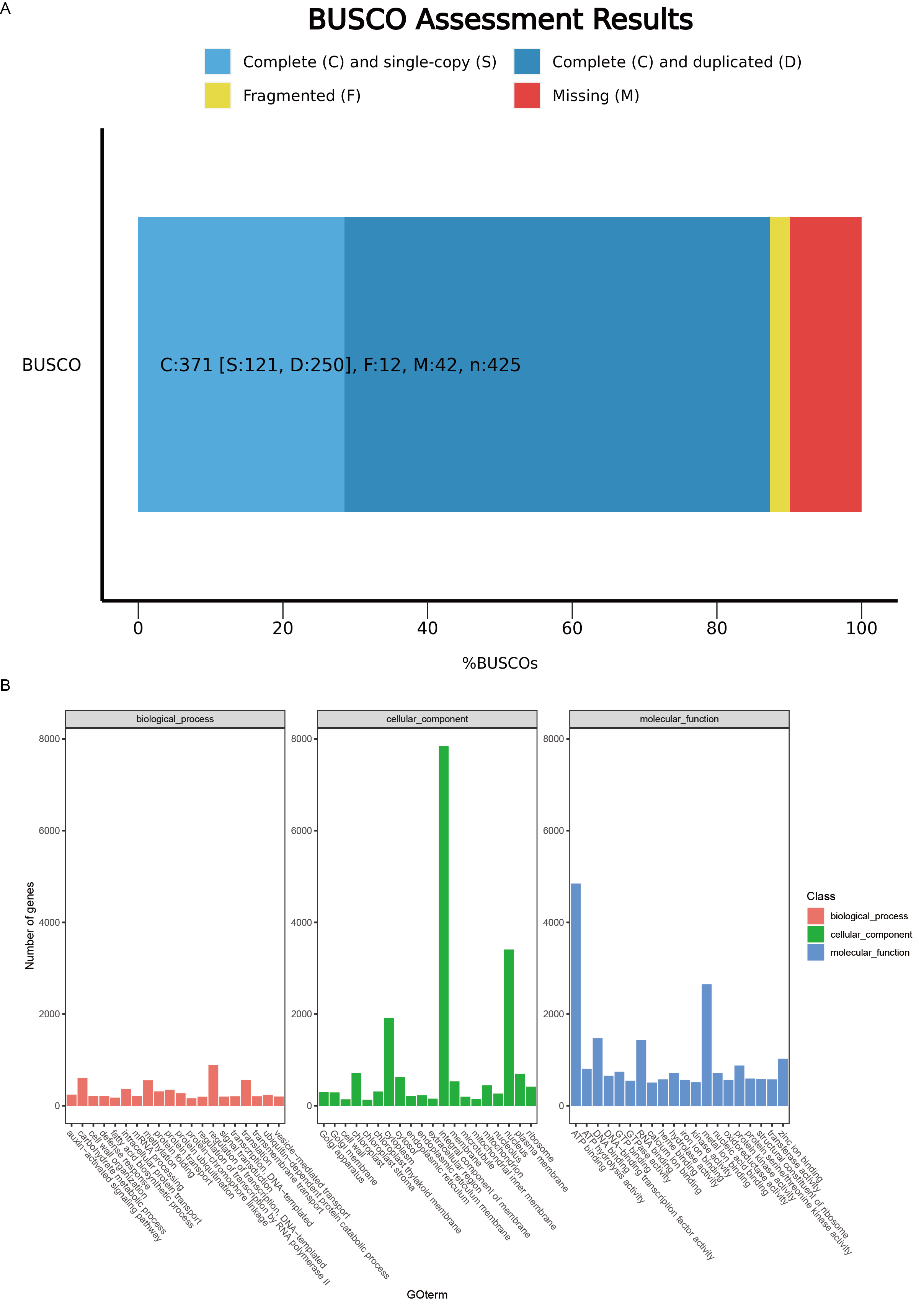


**Fig. S3** Analysis of *de novo* transcriptome. **a** BUSCO evaluation results of the *de novo* transcriptome of *S. yunnanensis.* **b** GO clustering analysis of the *de novo* transcriptome of *S. yunnanensis*.


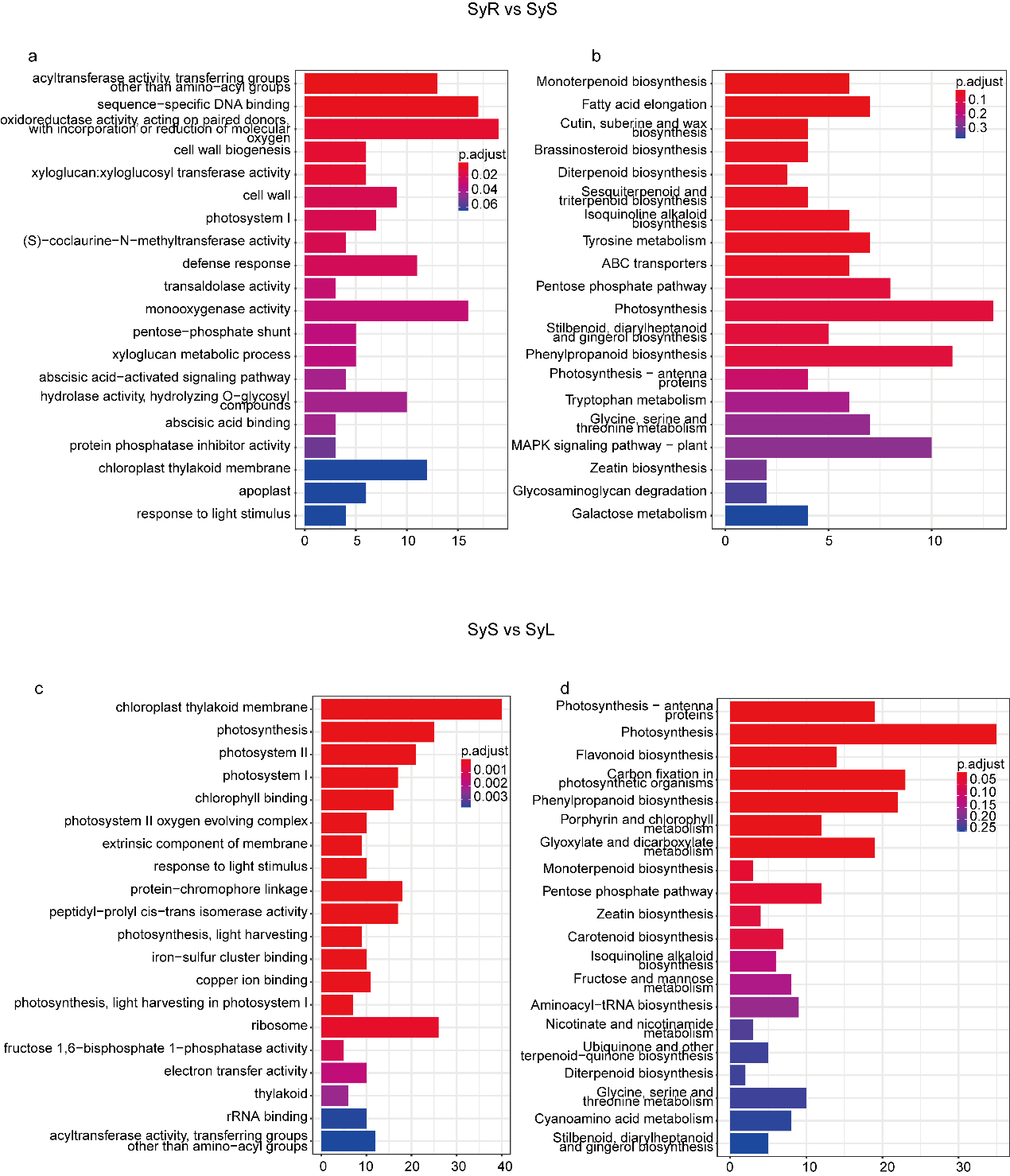


**Fig. S4** GO and KEGG pathway enrichment of DEGs of root vs. stem and stem vs. leaf comparisons. Here, SyR, SyS, and SyL represent the roots, stems, and leaves of *S. yunnanensis*.


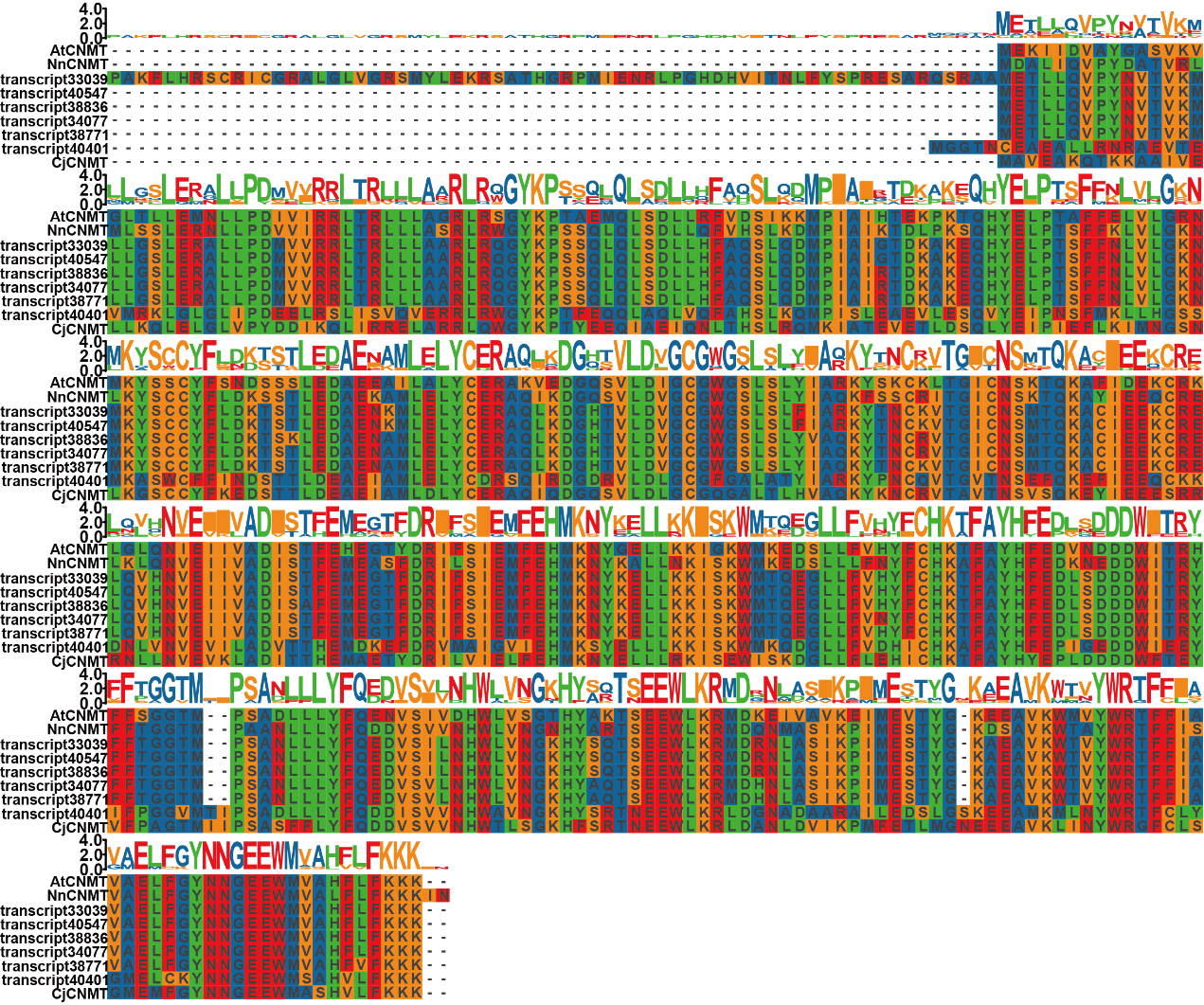


**Fig. S5** sequence alignment of candidate CNMTs and reference genes.


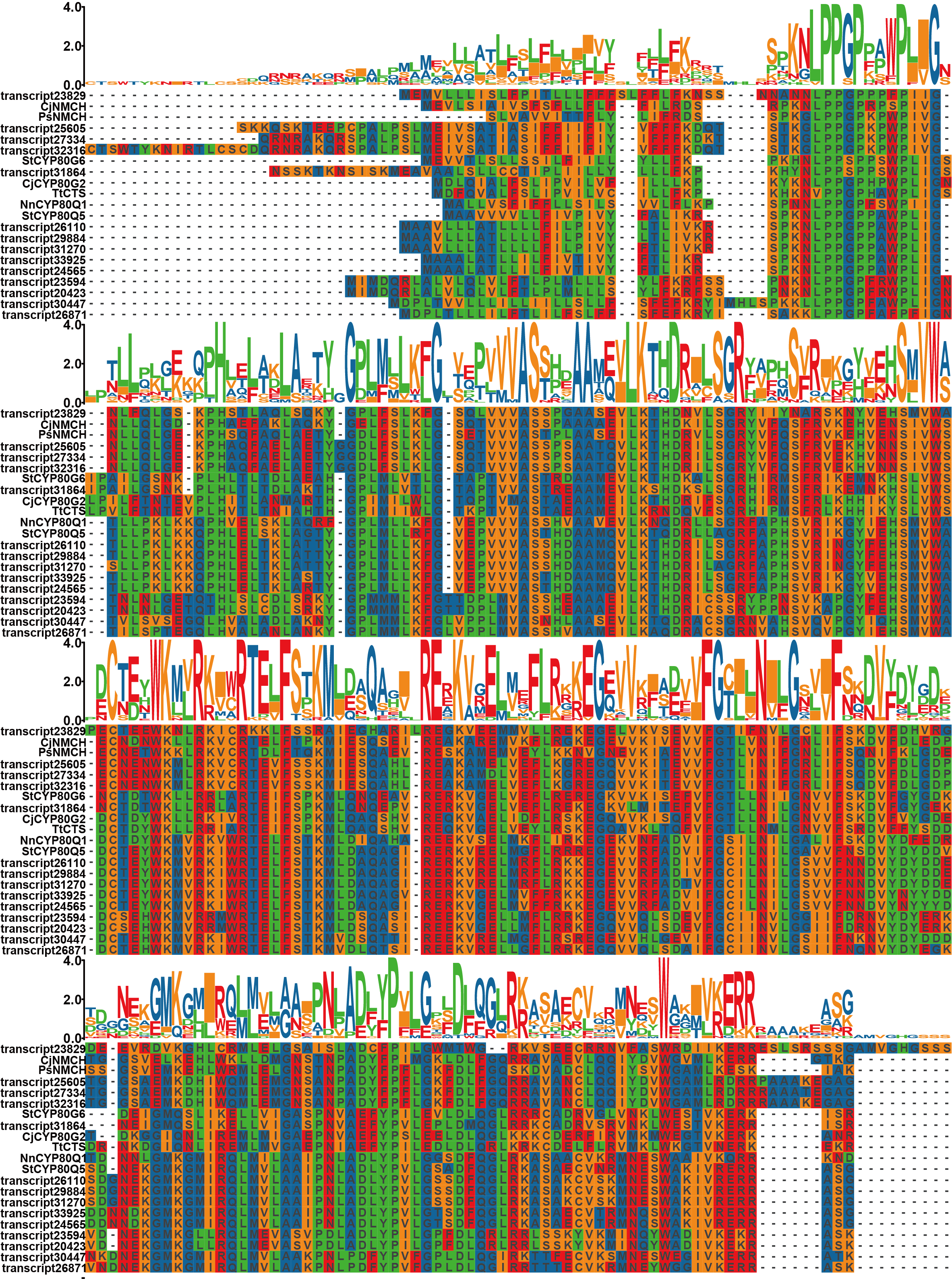


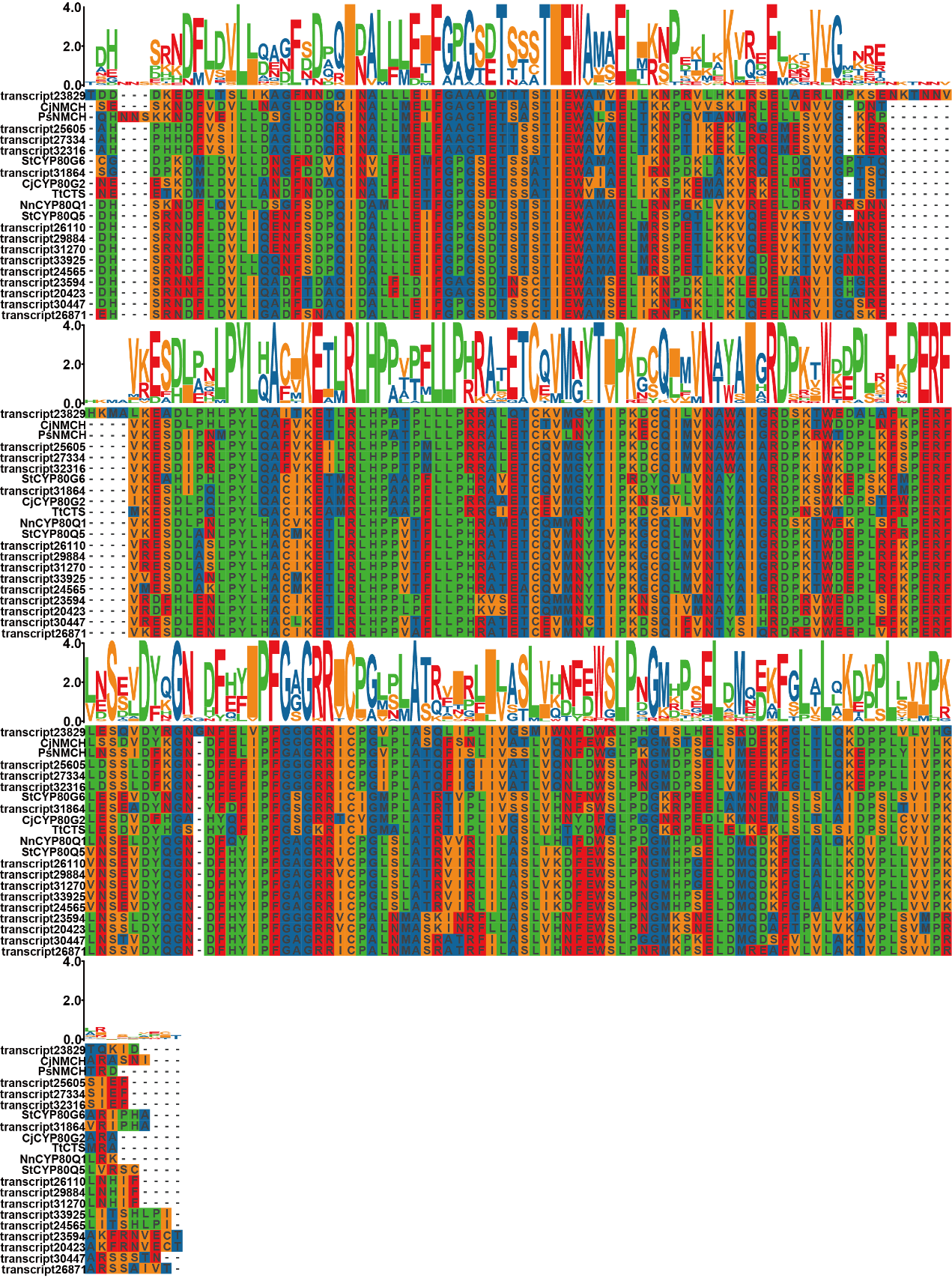


**Fig. S6** sequence alignment of candidate CYP80s and reference genes.


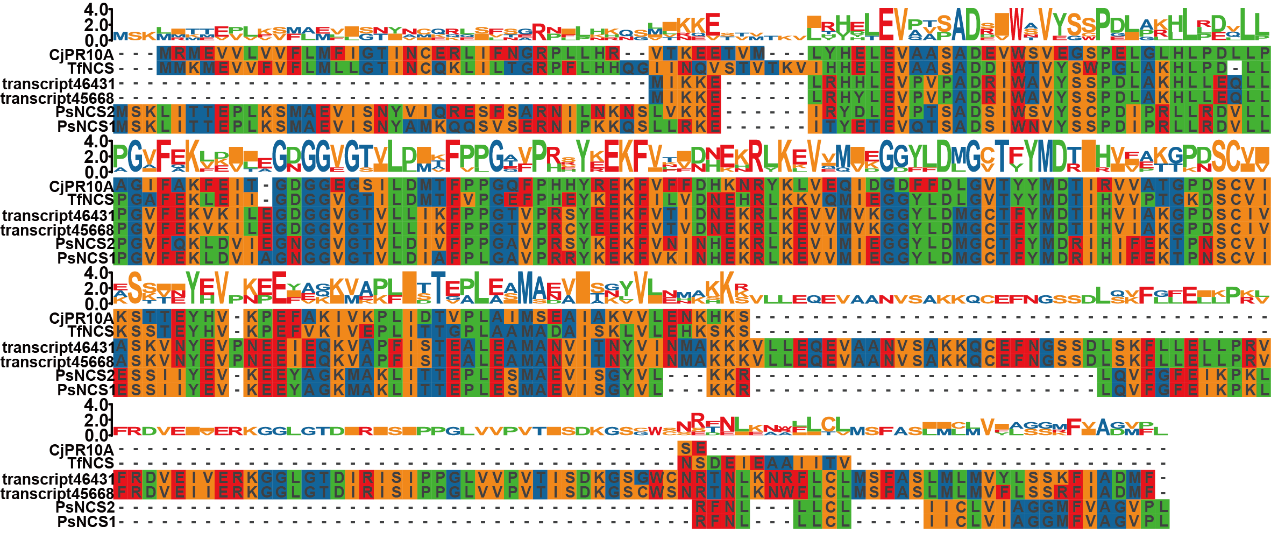


**Fig. S7** sequence alignment of candidate NCSs and reference genes.


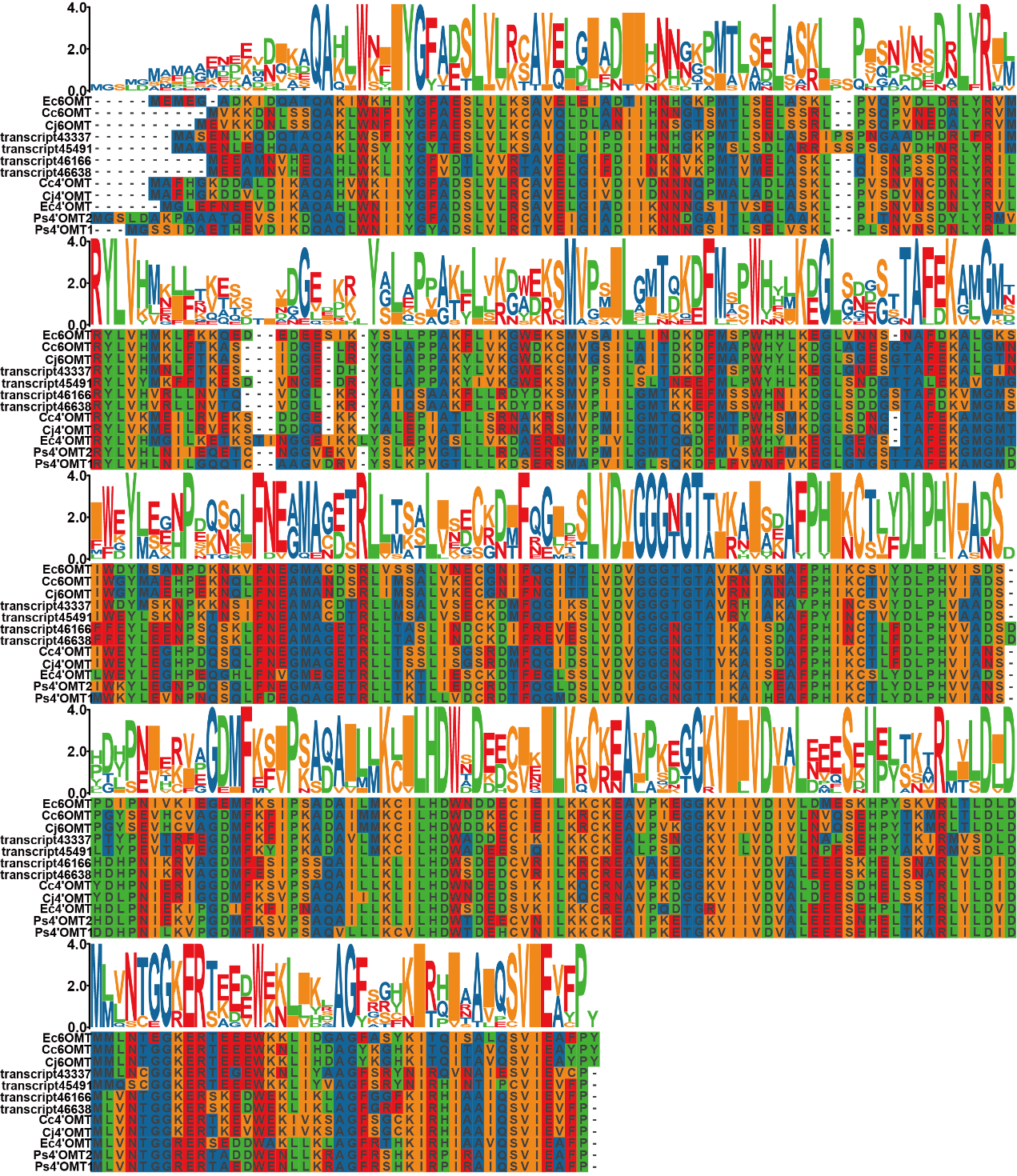


**Fig. S8** sequence alignment of candidate OMTs and reference genes.


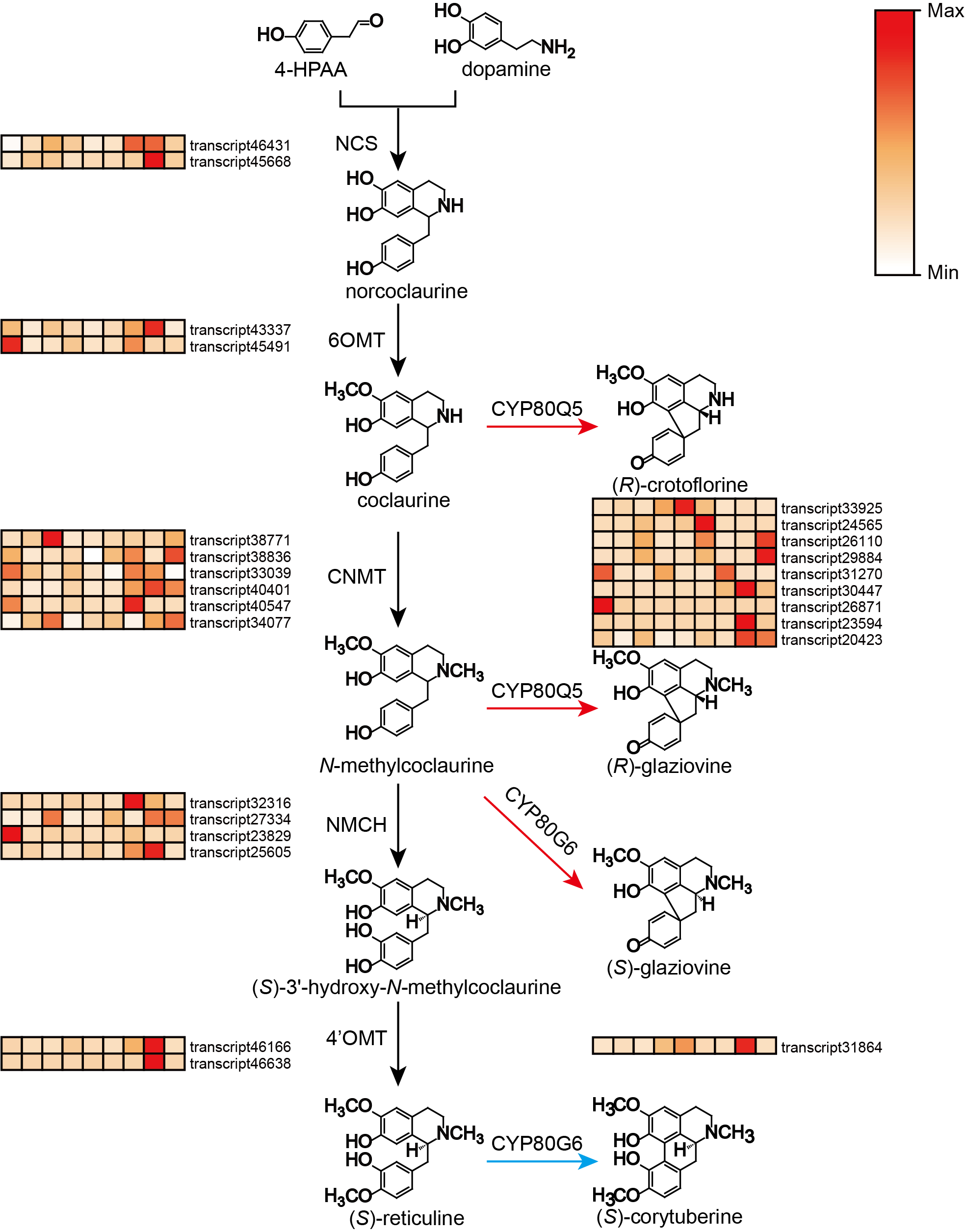


**Fig. S9** Expression patterns of candidate transcripts involved in the biosynthesis of BIAs in *S. yunnanensis*. Putative biosynthetic pathway for BIAs and analysis of gene expression patterns related to BIAs in *S. yunnanensis*. The expression levels in different samples are indicated by small squares in different colors, the colors indicate the expression levels, with blue representing low expression and red representing high expression. From left to right: leaf 1, leaf 2, leaf 3, stem 1, stem 2, stem 3, root 1, root 2, root 3. The pathway for type Ⅰ aporphines is highlighted in blue, while the pathway for type Ⅱ protoaporphines is highlighted in red.


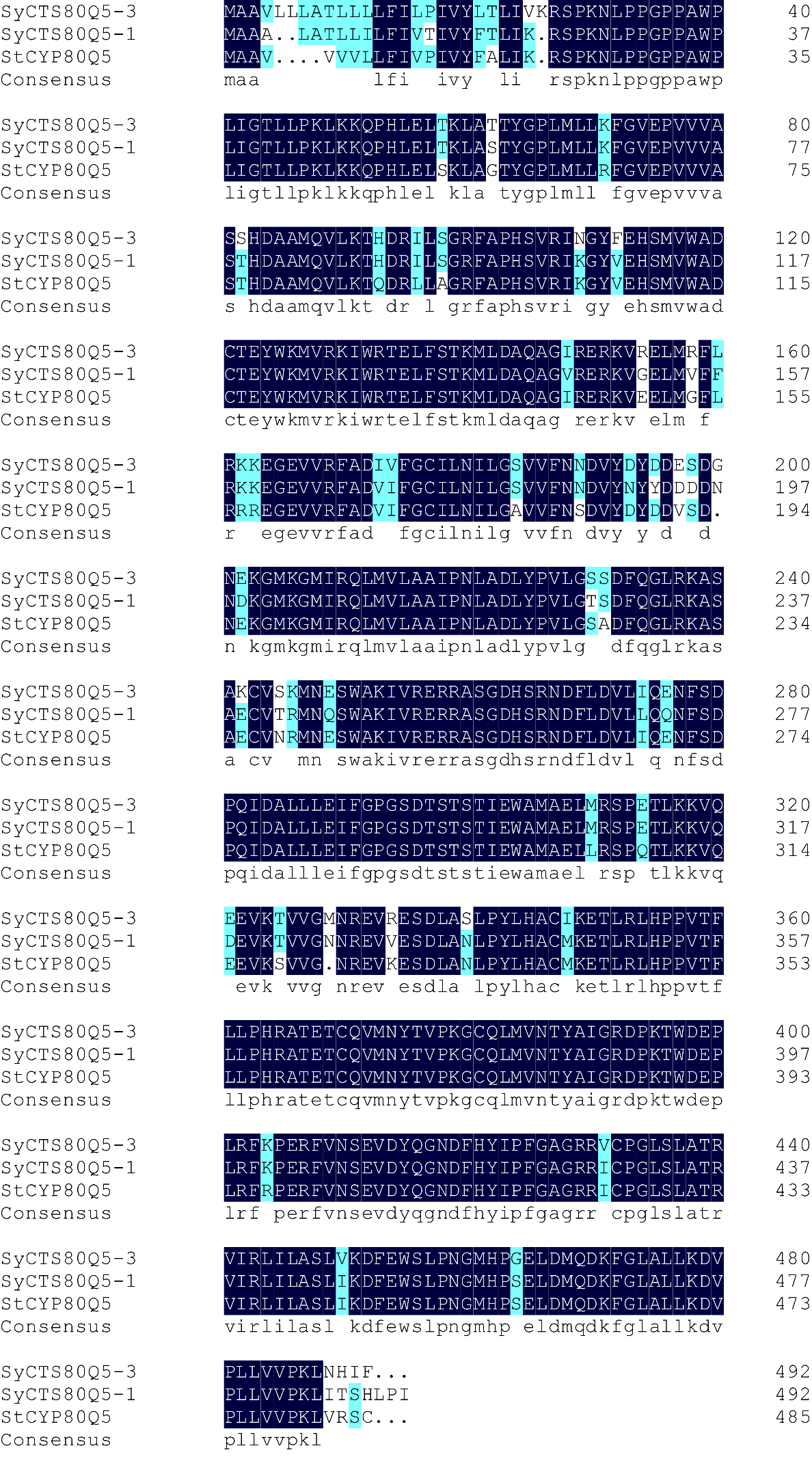


**Figure S10** Sequence alignment of SyCYP80Q5-3, SyCYP80Q5-1 and StCYP80Q5


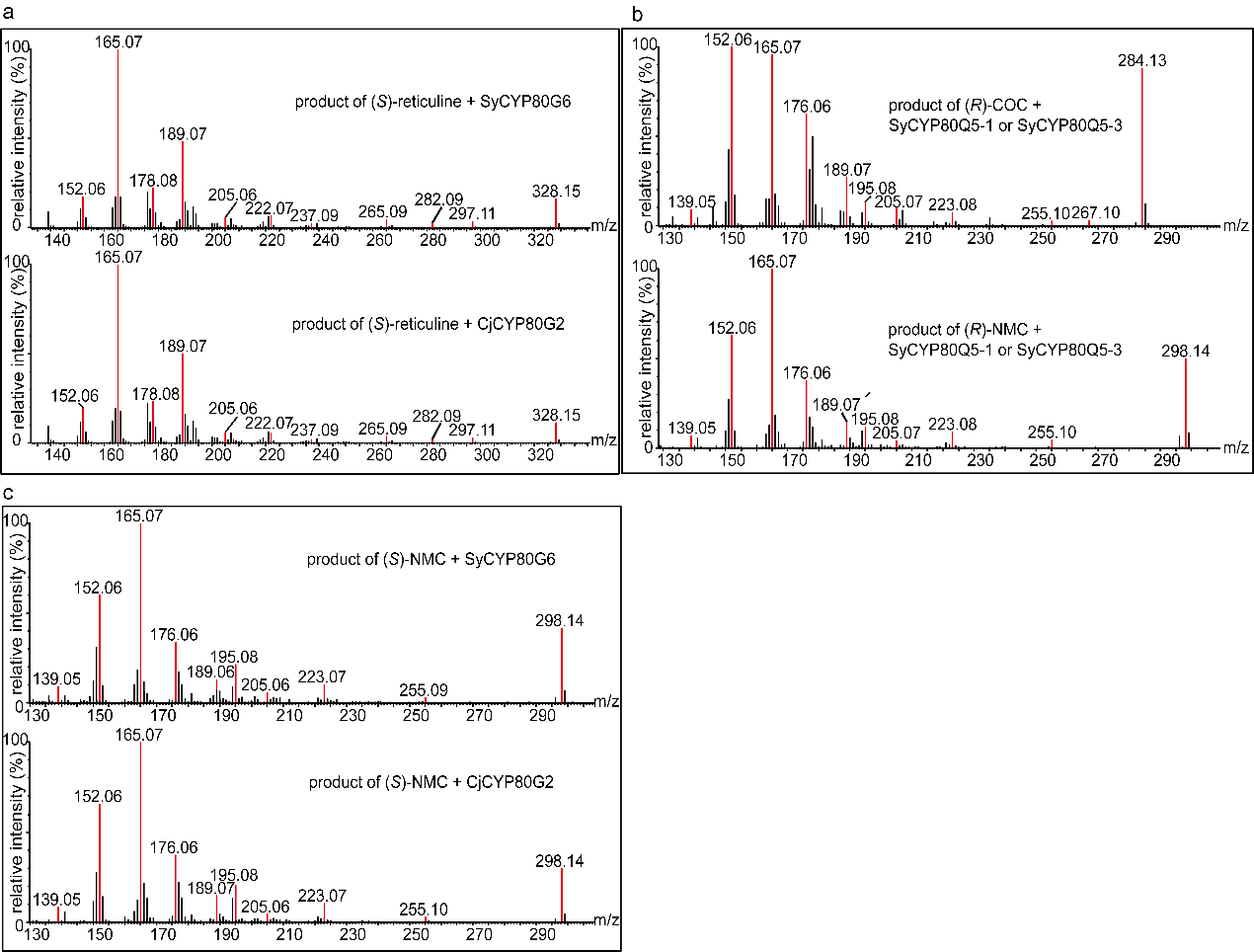


**Fig. S11** High-Resolution MS/MS spectrum Fragmentation of Products catalyzed by CYP80s, including **a** comparison between the products of SyCYP80G6 and CYP80G2 catalyzing (S)-reticuline, **b** comparison between the two produced of SyCYP80Q5-1 and SyCYP80Q5-3 catalyzing (*R*)-COC and (*R*)-NMC, and **c** comparison between the products of SyCYP80G6 and CYP80G2 catalyzing (*S*)-NMC


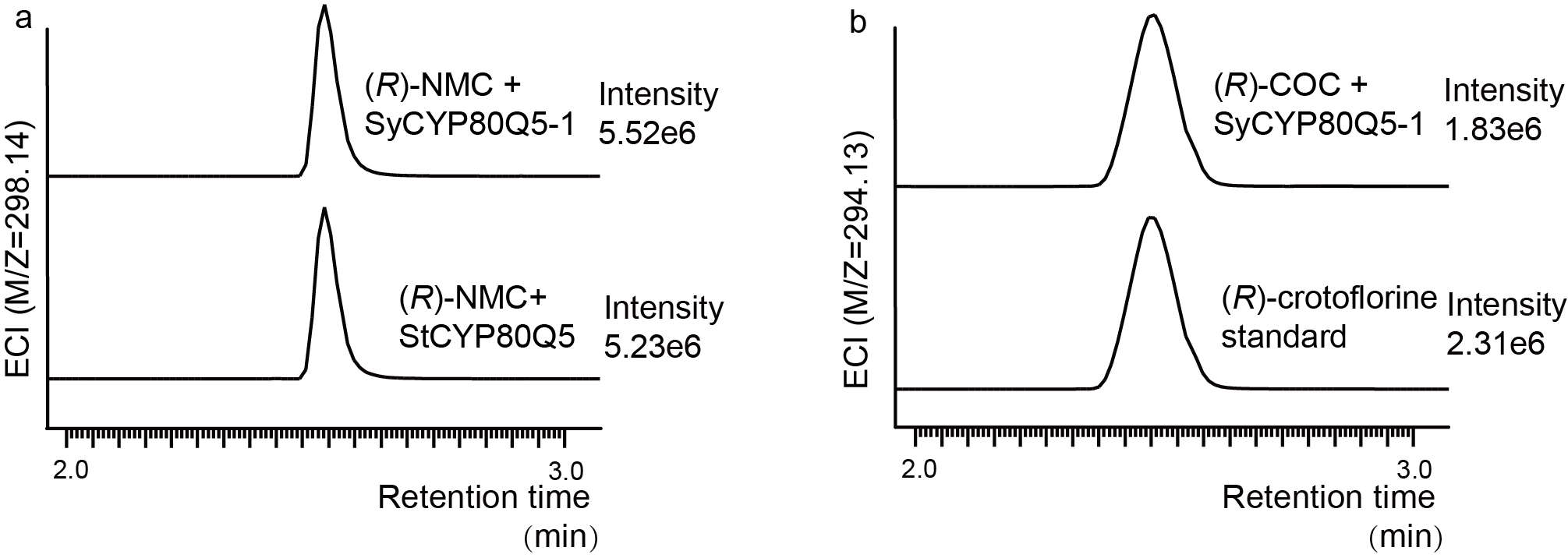


**Fig. S12** **a** comparison between the products of SyCYP80Q5-1 and StCYP80Q5 catalyzing (*R*)-NMC, and **b** comparison between (*R*)-crotoflorine standard and the products of SyCYP80Q5-1


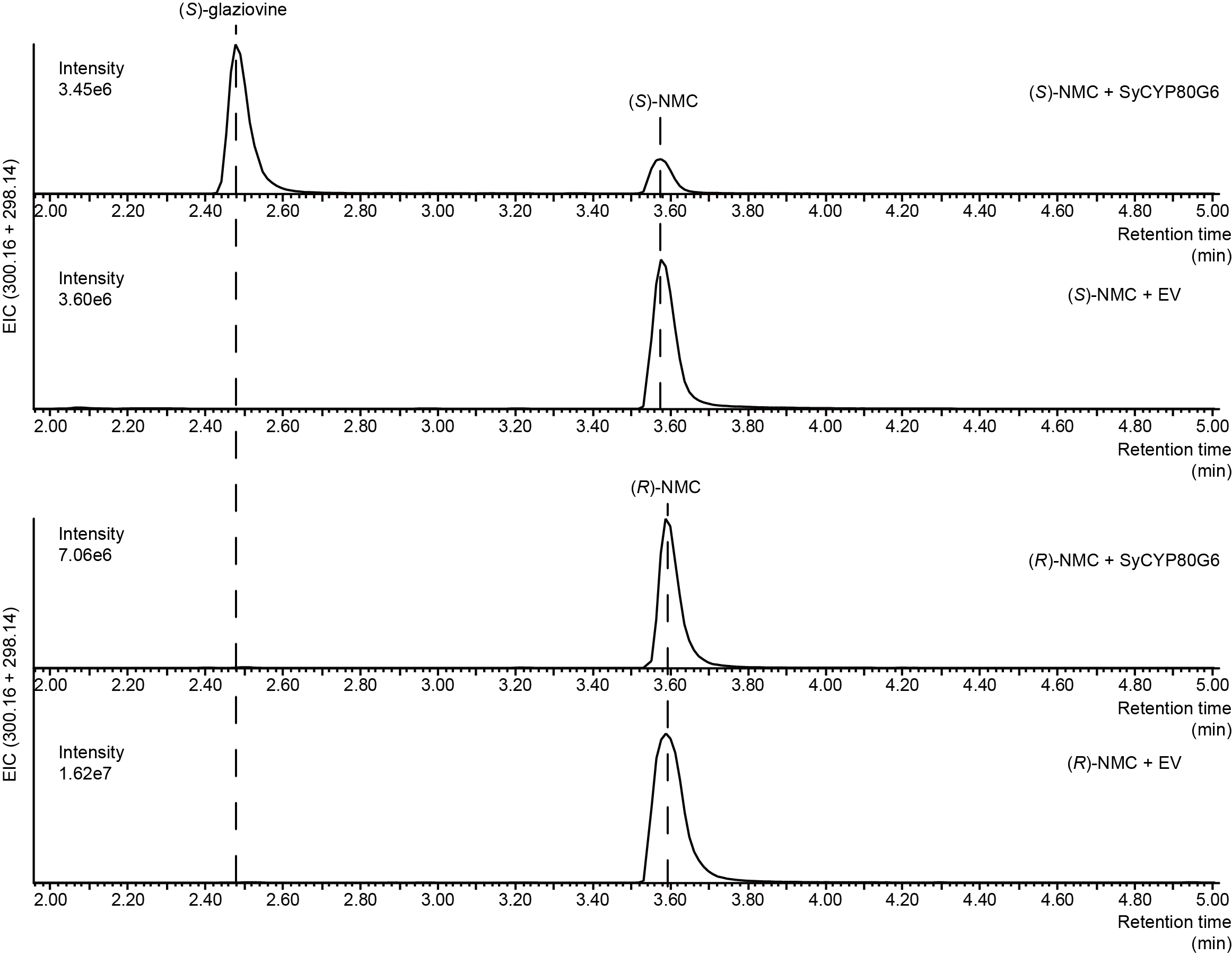


**Fig. S13** Reaction of SyCYP80G6 with NMC.


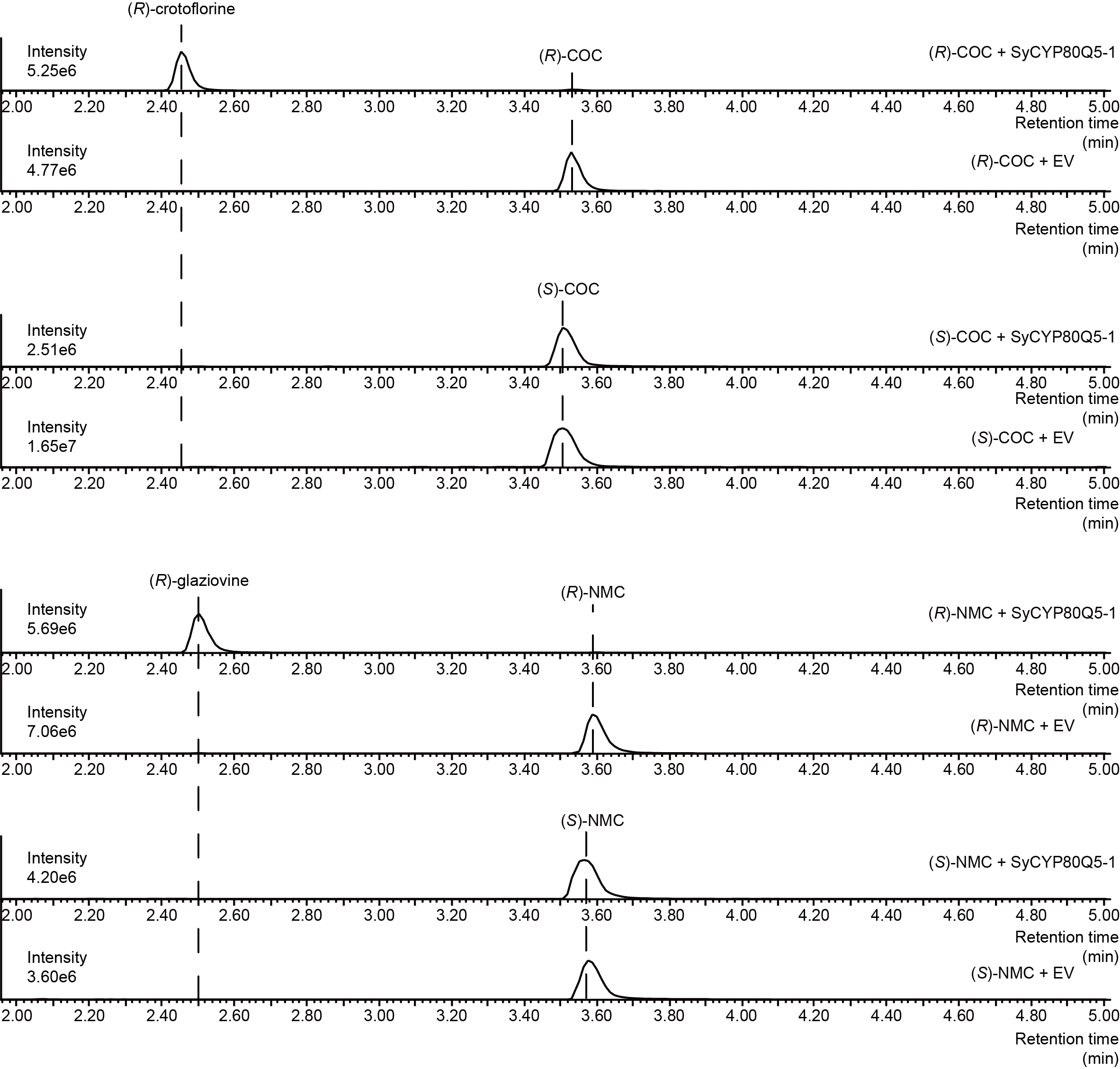


**Fig. S14** Reaction of SyCYP80G6 with NMC and COC.
